# An Induced Mutation in *HvRECQL4* Increases the Overall Recombination and Restores Fertility in a Barley *HvMLH3* Mutant Background

**DOI:** 10.3389/fpls.2021.706560

**Published:** 2021-11-12

**Authors:** Mikel Arrieta, Malcolm Macaulay, Isabelle Colas, Miriam Schreiber, Paul D. Shaw, Robbie Waugh, Luke Ramsay

**Affiliations:** ^1^Cell and Molecular Sciences, The James Hutton Institute, Dundee, United Kingdom; ^2^Information and Computational Sciences, The James Hutton Institute, Dundee, United Kingdom; ^3^Division of Plant Sciences, The University of Dundee at The James Hutton Institute, Dundee, United Kingdom

**Keywords:** meiosis, suppressor screen, recombination, barley, desynaptic mutant, crossover

## Abstract

Plant breeding relies on the meiotic recombination or crossing over to generate the new combinations of the alleles along and among the chromosomes. However, crossing over is constrained in the crops such as barley by a combination of the low frequency and biased distribution. In this study, we attempted to identify the genes that limit the recombination by performing a suppressor screen for the restoration of fertility to the semi-fertile barley mutant *desynaptic10* (*des10*), carrying a mutation in the barley ortholog of *MutL-Homolog 3* (*HvMLH3*), a member of the MutL-homolog (MLH) family of DNA mismatch repair genes. *des10* mutants exhibit reduced recombination and fewer chiasmata, resulting in the loss of obligate crossovers (COs) leading to chromosome mis-segregation. We identified several candidate suppressor lines and confirmed their restored fertility in an *Hvmlh3* background in the subsequent generations. We focus on one of the candidate suppressor lines, *SuppLine2099*, which showed the most complete restoration of fertility. We characterized this line by using a target-sequence enrichment and sequencing (TENSEQ) capture array representing barley orthologs of 46 meiotic genes. We found that *SuppLine2099* contained a C/T change in the anti-CO gene *RecQ-like helicase 4 (RECQL4)* resulting in the substitution of a non-polar glycine to a polar aspartic acid (G700D) amino acid in the conserved helicase domain. Single nucleotide polymorphism (SNP) genotyping of F_3_ populations revealed a significant increase in the recombination frequency in lines with *Hvrecql4* in the *Hvmlh3* background that was associated with the restoration of fertility. The genotyping also indicated that there was nearly double the recombination levels in homozygous *Hvrecql4* lines compared to the wild type (WT). However, we did not observe any significant change in the distribution of CO events. Our results confirm the anti-CO role of *RECQL4* in a large genome cereal and establish the possibility of testing the utility of increasing recombination in the context of traditional crop improvement.

## Introduction

The crossing over that occurs during meiosis generates new combinations of the alleles that subsequently become the substrate for the selection either *via* the natural evolution or by human-driven selection during the traditional plant breeding. When crossing over occurs in a hybrid plant, genetic materials are physically exchanged among the homologous chromosomes with the resulting progeny containing a genotypic mosaic of the original hybrid genome. In an inbreeding crop plant such as barley, subsequent generations of the self-fertilization and selection return the plants to near homozygosity and it is these genotypes that are further multiplied and commercially marketed as the fixed inbred lines or cultivars. Barley has a large genome of around 5 Gb (Mascher et al., 2017; Jayakodi et al., 2020; Schreiber et al., 2020) and similar to the other crops, the number and distribution of crossovers (COs) are limited (Blary and Jenczewski, 2019). In particular, CO position is highly skewed to the distal regions of the chromosomes, which creates recombination-poor regions of the genome that contain a considerable number of genes (Mayer et al., 2012; Darrier et al., 2017; Mascher et al., 2017). This lack of recombination impacts genetic variation, constrains breeding progress and the introgression of the novel traits, and reduces the efficiency of molecular cloning and the discrimination of quantitative trait locus (QTL) (Darrier et al., 2017; Blary and Jenczewski, 2019). It has been proposed that positively increasing recombination could improve the speed and accuracy of plant breeding and genetic programs (Wijnker and de Jong, 2008).

Crossovers arise from the repair of double-strand breaks (DSBs) in DNA (Wang and Copenhaver, 2018) via two alternative pathways. Class I is the predominant pathway that results in homologous recombination (HR). It is considered to be sensitive to interference, i.e., the existence of one CO limits the formation of another one close by each other (Berchowitz and Copenhaver, 2010). Class I repairs are dependent upon the well-studied ZMM (ZYP, MSH, and MER-3) protein complexes and the pro-CO E3 ligase HEI10 and involve the DNA mismatch repair proteins such as MutL-homologs (MLH1 and MLH3) (Mercier et al., 2015; Wang and Copenhaver, 2018). Class II COs are generated by the CO junction endonuclease MUS81-dependent pathway (Higgins et al., 2008; Wang and Copenhaver, 2018). These are believed to be mostly insensitive to interference and, thus, class II COs may occur relatively independently from each other. The frequency of class II COs has been estimated to be in the range of 15–25% of the total COs in *Arabidopsis* (Copenhaver, 2003; Mercier et al., 2005; Higgins et al., 2012) and 5–10% in barley (Barakate et al., 2014).

Genetic suppressor screens of *Arabidopsis* mutants (ZIP1, MSH4/5, MER3, SCHO1, and Hei10) with reduced CO and associated semi-sterility have identified genes repressing CO number by the restoration of fertility in the mutants such as anti-CO genes including the orthologs of *Fanconi anemia group M* (*FANCM)* (Crismani et al., 2012), *Fidgetin-like 1* (*FIGL1)* (Girard et al., 2015; Fernandes et al., 2018), *DNA topoisomerase 3*α (*TOP3*α) (Séguéla-Arnaud et al., 2015), and *RecQ-like helicase 4* (*RECQL4*) (Séguéla-Arnaud et al., 2015).

Following the principles of these *Arabidopsis* studies, we performed a suppressor screen by mutagenizing the semi-sterile barley mutant *desynaptic10* (*des10)* and then examining the individual progenies in the M_4_ generation for the restoration of fertility. *des10* (Lundqvist et al., 1997) was previously characterized as a 159-bp deletion in the mismatch repair gene *MutL-Homolog 3* (*HvMlh3*) and shows a much reduced number of COs, abnormal synapsis progression, chromosome mis-segregation, and a subsequent reduction of fertility (Colas et al., 2016). To the best of our knowledge, a suppressor screen of *MLH3* mutants has not been performed before in *Arabidopsis* perhaps because the phenotype is less severe compared to the ZMM mutants used (Crismani et al., 2012; Girard et al., 2015; Séguéla-Arnaud et al., 2015). Our phenotypic screen identified several candidate suppressor lines that improve fertility in the *Hvmlh3* genetic background. We focus on the phenotypic, molecular, and cytological characterization of the single line, *SuppLine2099*, which exhibited the most complete restoration of fertility.

## Materials and Methods

### DNA Extraction

Leaf tissue was collected from the seedlings ~2 weeks after sowing at the two-leaf stage of development. DNA extractions were carried out by using the QIAamp 96 DNA QIAcube HT Kit (Qiagen, Venlo, Netherlands) from ~100 mg of young leaf tissue as per the instructions of the suppliers on the QIAcube HT nucleic acid purification platform (Qiagen, Venlo, Netherlands).

### Suppressor Screen on *desynaptic10*

The suppressor screen used the Bc_5_F_5_ near-isogenic line BW230 *des10* (syn. *HvMlh3*) in the *cv*. Bowman background (Druka et al., 2010; Colas et al., 2016). BW230 *des10* plants were multiplied in a polytunnel and glasshouse to finally generate around 18,000 seeds. These seeds were divided into three separate lots and were mutagenized with Ethyl methanesulfonate (EMS) at 20, 25, and 30 mM concentrations, respectively, as described previously (Caldwell et al., 2004; Schreiber et al., 2019). M_1_ seeds were grown in summer 2016 in the soil in a polytunnel in Dundee (UK), harvested, and bulked by treatment (EMS concentration). This process was repeated for M_2_ and M_3_ generations (outline of the process in Supplementary Figure 1). Finally, 10 plants that showed increased fertility relative to the semi-sterile BW230 *des10* were selected from the M_4_ generation. These were genotyped with a custom Kompetitive allele-specific PCR (KASP) marker diagnostic for the presence/absence of the 159 bp deletion in *Hvmlh3* to confirm the presence of the *des10* mutation (Colas et al., 2016). A custom target enrichment MyBaits Array (Daicel Arbor Biosciences, Ann Arbor, Michigan, USA) comprising 46 meiotic genes was then used to screen for mutations, exactly as described previously (Schreiber et al., 2019).

### Plant Material

Plants (*Hordeum vulgare* L.) were grown in 6-inch pots in a glasshouse under 16 h of light at 18–20°C and 8 h of dark at 16°C. A general-purpose mix compost containing peat, sand, limestone, Perlite, Celcote, Osmocote®, and Exemptor® was used in all the experiments.

### Crossing and F_1_ Population Development

Crosses were made in the glasshouse with pollen from the barley *cv*. Barke used to pollinate the emasculated spikes of the suppressor line *SuppLine2099* (*cv*. Bowman background). F_1_ seeds were harvested and sown in 96 well single seed descent (SSD) trays in the glasshouse, where leaf tissue was collected and DNA extracted as described above. The success of the cross was confirmed by checking F_1_ plant heterozygosity by genotyping the plants with a series of custom made KASP (LGC) markers (Supplementary Material 1A) and subsequent sequencing of the alleles. Confirmed F_1_ plants were moved to 6-inch pots and grown in the glasshouse (bagged at flowering) to produce a large number of self-fertilized F_2_ seeds forming a segregating F_2_ population. An outline of the experiment is illustrated in Supplementary Figure 2.

### F_2_ Population Development

F_2_ seeds from a single F_1_ line were sown in 96 well SSD trays in the glasshouse. After DNA extraction, a total of 267 F_2_ plants from the suppressor line cross were genotyped for the mutations in the two target genes by KASP markers (Supplementary Material 1B). Genotyping allowed the identification of the four groups of F_2_ plants that were homozygous at both the loci for either mutant or the wild type (WT) allele (Supplementary Figure 2). A total of 52 plants were selected and those plants were moved to bigger 6-inch pots in the glasshouse conditions to produce the F_3_ seeds to be genotyped with the 50K iSelect single nucleotide polymorphism (SNP) array (Bayer et al., 2017).

### F_2_ Fertility Assessment

The fertility of the selected F_2_ plants was assessed by counting the fertile seeds and total florets on three random ears per mature plant and by threshing all ears of each plant with a single ear thresher (Haldrup GmbH, Ilshofen, Germany) and calculating the thousand grain weight (TGW), grain weight per ear, and seeds per ear with a Marvin Digital Seed Analyser (GTA Sensorik GmbH, Neubrandenburg, Germany). Fertility comparisons were carried out by using a *t*-test with the *ggplot2* (https://CRAN.R-project.org/package=ggplot2) and *ggpubr* (https://CRAN.R-project.org/package=ggpubr) packages in R software (version 3.6.1, R Foundation, Vienna, Austria).

### F_3_ Population Development

The 52 selected F_2_s were genotyped by using the 50K iSelect SNP array and 10 individuals from each of the four homozygous allele groups were chosen to maximize the informative heterozygous genome coverage in the final F_3_ populations to be used for the genetic analysis. F_3_ seeds from chosen F_2_ family were sown in 96 well SSD trays in the glasshouse, aiming to grow 10 F_3_ plants per chosen F_2_ family (one family would remain smaller). DNA was extracted from these plants (for 50k genotyping and recombination analysis) and once mature, F_4_ seeds were harvested for the cytological analysis.

### Kompetitive Allele-Specific PCR Genotyping

Diagnostic KASP assays were designed for each of the two target mutations segregating in the F_2_ families (Supplementary Material 1B). DNA sequence containing 70 bp on each side of the mutations was used to design two allele-specific and a common primer for each KASP assay with a web-based allele-specific primer (WASP) design tool (https://bioinfo.biotec.or.th/WASP). Primers were synthesized by Sigma (Sigma-Aldrich Co Ltd (Merck), Kenilworth, NJ, USA). Eight μl reactions were prepared in the MicroAmp Fast optical 96-well-plates (Thermo Fisher Scientific, Waltham, MA, USA) by using <3 ng of DNA, 4 μl of 2 × KASP reagent (LGC, Middlesex, UK), two allele-specific primers at 0.16 μM each, and a conserved primer at 0.4 μM. PCR and genotyping were completed by using a StepOne Plus real-time PCR machine (Applied Biosystems, Thermo Fisher Scientific, Waltham, MA, USA). Sample fluorescence was measured at 20°C for 2 min; then, DNA was denatured for 15 min at 94 C followed by 10 cycles of 20 s at 94 C and 1 min at 62 C (decreasing by 0.7 C per cycle). This was followed by 32 cycles of 20 s at 94 C and 1 min at 55 C. Samples were then cooled to 20 C for 2 min to allow the fluorescence measurement. Alleles were scored by using the StepOnePlus Software (Applied Biosystems, Thermo Fisher Scientific, Waltham, MA, USA).

### 50K Genotyping and Scoring

DNA was quantified and quality checked by using a spectrometer (NanoDrop Technologies LLC, DE, USA). DNA with absorbance ratios at both 260/280 nm and 260/230 nm of > 1.8 was used and the DNA concentration was adjusted by dilution to 20 ng/μl. A total amount of 300 ng DNA per sample was lyophilized and sent to the Geneseek (Neogen Europe, Ltd., Auchincruive, UK) for Illumina high-throughput screening by using the Infinium HTS assay and the HiScan array imaging (Illumina, San Diego, California, USA) by using the Barley 50K iSelect SNP array (Bayer et al., 2017). R and Theta scores were extracted from the resulting idat files by using the GenomeStudio Genotyping Module version 2.0.2 (Illumina, San Diego, California, USA) and the allele scores were created by using the paRsnps (an in-house software package for clustering, visualizing, and comparing the Illumina SNP genotyping data).

### 50K Data Cleaning and Recombination Analysis

For the preliminary 50K data cleaning, a custom R script was used to remove the monomorphic and ambiguous markers showing highly skewed allele frequencies. The latter SNP data were characterized by intercalated isolated heterozygous calls which falsely inflated the number of the COs, so individuals with an abundance of such calls leading to CO number higher than 35 were discarded from the analysis. Markers with > 5% missing data were also removed. The nucleotide calls were then changed to A/B/H format with A being the homozygous allele call for the *SuppLine2099* parent B for *cv*. Barke parent and H for a heterozygous allele call.

The physical order of the markers and their respective physical position on the barley genome was obtained from the current physical assembly (MorexV2, Monat et al., 2019). Prior to CO scoring, for each F_3_ family (sharing the same F_2_ origin), markers that were homozygous in the parental F_2_ individual were removed from the analysis, so that only recombination in the F_2_ would be assessed. CO events were scored as an allele change between the two consecutive markers; a single CO was considered when either the homozygous allele call of any of the two parental alleles changed to a heterozygous call or vice versa. Changes from either the parental homozygous call to the other were considered as a double CO. In order to consider any CO as validated, the allele change needed to be maintained in the following three markers to the position of the switch. This rather conservative approach may underestimate the number of COs, but avoided the inflation of the CO counts by the isolated false allele calls. The effect of missing data in the three-marker window on CO calling was corrected manually.

Recombination fraction (r) was calculated by dividing the number of COs between the two markers and the number of the individuals that had no missing data for those two markers. The recombination distance was then calculated by using the Kosambi map function:


$$\begin{array}{l} Kosambi\;distance\;\left(cM\right)\;=\;\frac{ln\left[\left(1+2*\left(\frac{r}{2}\right)\right)/\left(1-2*\left(\frac{r}{2}\right)\right)\right]}{4}*100 \end{array}$$

The mid-physical point between the two markers was used as the physical position of a CO for binning recombination events in the physical intervals and *ggplot* and *ggpubr* R packages were used to plot the recombination frequency and distribution graphs.

Differences in recombination between the four homozygous allele groups for entire chromosomes and for the three chromosomal zones described in Mascher et al. (2017) were compared using the Wilcoxon's signed rank test (as in Devaux et al., 1995) using the R *ggpubr* package. The comparison of the relative proportion of recombination was carried out with the contingency table and the chi-squared test.

### Cytology

F_4_ seeds from randomly chosen F_3_ families were grown under controlled standard conditions (16 h light at 18°C, 8 h dark at 14°C) in 6-inch pots. Spikes of between 1.8 and 2.2 cm (at metaphase stage) were harvested from the plants and fixed in 1:3 acetic acid/EtOH for at least 24 h at 4–6°C. Anther dissection was carried out under a stereo microscope (Leica Microsystems, Wetzlar, Germany) and squashed on a microscope slide in a drop of 0.5% acetocarmine. Meiotic stages were determined by checking the slides under light microscope (Olympus K2, Olympus Corporation, Tokyo, Japan) and chromosome spreads were prepared according to Colas et al. (2016) with slight modification. Slides were mounted in Fluoroshield containing 0.0002% 4′,6-diamidino-2-phenylindole (DAPI) (Abcam, ab104139, Cambridge, UK), and the coverslips were sealed with nail polish. Three-dimensional (3D) stack images (512 × 512, 12 bits, optimal sectioning) were taken with a confocal Zeiss LSM 710 microscope equipped with the W Plan-Apochromat 63X/1.0 M27 objective (laser light 405 nm, 4 lines averaging). Raw images were processed with the Imaris Deconvolution ClearView 9.5 (Imaris, Zurich, Switzerland) (parameters in Supplementary Material 2). Chiasmata were manually counted by using the Imaris 9.5.1 (Imaris, Zurich, Switzerland) Spot module from the stack images. Projections of 3D pictures, Gaussian smoothing, and light brightness/contrast/color adjustments were performed with the Imaris 9.5.1 (Imaris, Zurich, Switzerland) (Bitplane).

## Results

### Suppressor Screen of BW230 *desynaptic10*

18,000 seeds of BW230 *des10* (syn. *Hvmlh3*) (Colas et al., 2016) were mutagenized with 20, 25, and 30 mM EMS and grown in a polytunnel in 2016. M_2_ plants and M_3_ families were grown again in the same facility in 2017 and 2018, respectively, and screened for increased fertility when compared to BW230 *des10*. 10 M_4_ plants were identified and 3 M_5_ plants from each were grown in the glasshouse to confirm the fertility levels (Supplementary Figure 3). To explore whether the suppressor lines contained mutations in any previously characterized meiotic genes, we conducted a sequence-based screen by using a hybridization-capture MyBaits probe pool (Schreiber et al., 2019). The capture contained probes covering the exonic regions of the barley orthologs of 46 known meiotic genes including the anti-CO genes discovered previously in *Arabidopsis* (Schreiber et al., 2019) with some intronic and untranslated region (UTR) sequences also captured due to gene structure and the length of reads (Schreiber et al., 2019). Hybridization capture followed by sequencing on an Illumina MiSeq instrument revealed that nine of the fertility-restored plants contained mutations in the different meiotic genes (Supplementary Table 1). In this study, we focus on one line, *SuppLine2099*, which showed the highest level of the restored fertility compared to BW230 *des10* (Supplementary Figure 3).

**Figure 1:**
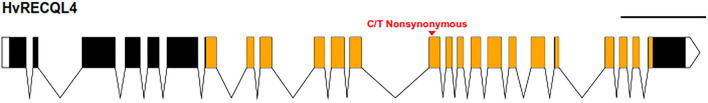
Graphical representation of *HvRECQl4*. The 24 exons are represented by the boxes joined by the introns with untranslated regions (UTRs) unfilled and the conserved helicase domain in orange. The position of the mutation is marked by the red arrow. Scale bar: 1 kb. Diagram produced with the exon-intron graphic maker (wormweb.org/exonintron).

Mapping the Illumina reads against the genomic sequence of the 46 meiotic genes used in the probe pool (Schreiber et al., 2019) revealed that *SuppLine2099* contained a non-synonymous C/T point mutation (Figure 1) in the coding sequence of *HvRECQL4* (CDS included in Supplementary Material 3); the C/T change in exon 13 resulted in a predicted amino acid change from a non-polar glycine to a polar aspartic acid (G700D) amino acid in the conserved helicase domain. Analysis of the likely impact of this substitution using the Protein Variation Effect Analyzer (PROVEAN) (Choi et al., 2012) revealed a score of 6.851, which is predicted as deleterious.

### A Mutation in *RecQ-Like Helicase 4* Restores Fertility to BW230 *desynaptic10*

**Figure 2:**
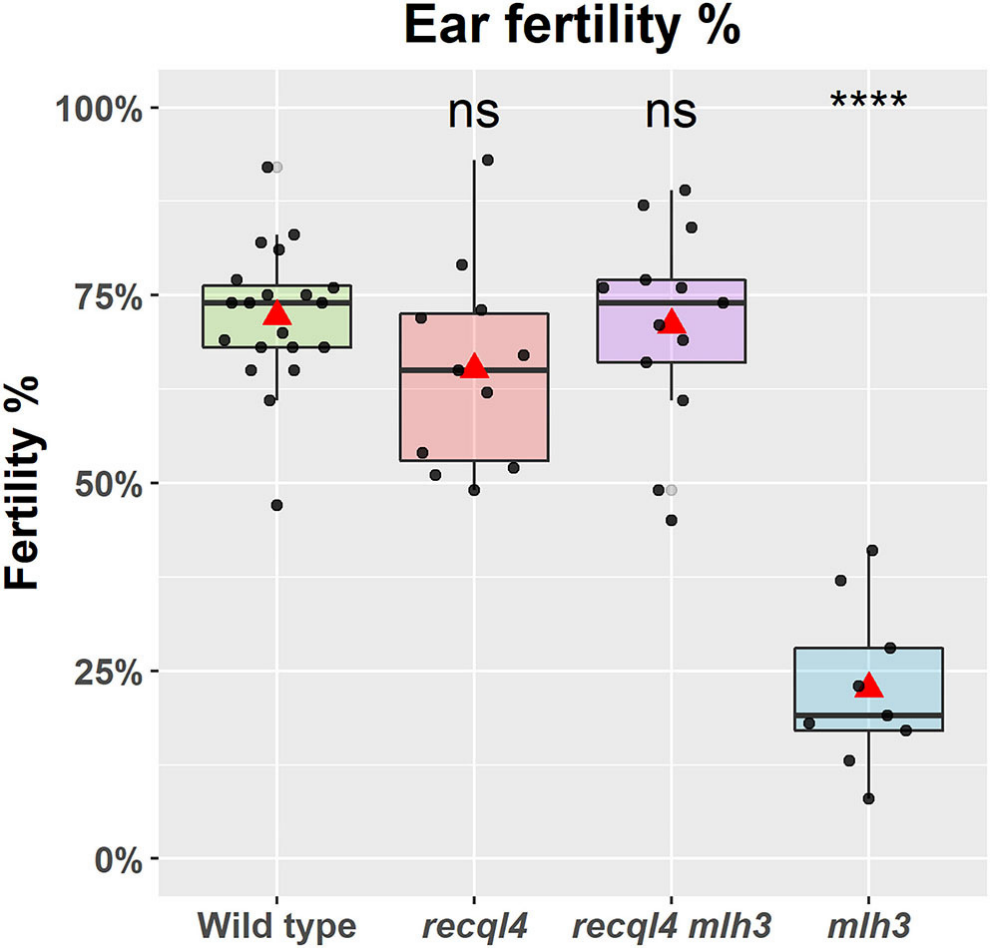
Fertility of the selected F_2_ plants as a percentage of fertile florets per ear (%). The statistical comparison with the wild type (WT) was done with the *t*-test where “ns” is not significant, ****p < 0.0001. The red triangle shows the mean per genotype. Each dot in the graph represents the average three ears of one plant.

To confirm that the G700D substitution in HvREQL4 was responsible for the restoration of fertility, *SuppLine2099* was crossed onto *cv*. Barke and F_1_ plants grown to generate F_2_ seed (outline of the experiment shown in Supplementary Figure 2). F_2_ plants were then selected based on their genotype at *HvRECQL4* and *HvMLH3*, with the seed of the following four possible homozygous classes identified and selected: *RECQL4 MLH3, recql4*_G700D_ *MLH3, RECQL4 mlh3*_*des*10_, or *recql4*_G700D_ *mlh3*_*des*10_, which will be referred to as the WT, *recql4, mlh3*, and *RECQL4 MLH3* (double mutant), respectively. The number of plants in each class was 20, 11, 9, and 13, respectively. The fertility of the F_2_s was assessed in the glasshouse by using three ears of each plant. A significant (*p* < 0.0001) reduction of fertility (Figure 2) was observed in the *mlh3* plants, which had an average of 22.3% of fertile seeds/ears compared to the WT with 72.2%, while the *recql4* and *RECQL4 MLH3* classes showed no significant difference to the WT (65.2 and 71.1%). The restitution of fertility in the *RECQL4 MLH3* class was clear with similar fertility to the *recql4* class, despite slight differences in the number of seeds and number of florets (Supplementary Figure 4). No differences were found between the four F_2_ subpopulations for TGW suggesting that once seed had set neither mutations individually nor together had a significant impact on the seed development (Supplementary Figure 4).

### *Hvrecql4_*G*700*D*_* Mutation Restores and Increases the Number of Chiasmata in a *Hvmlh3_*des*10_* Background

**Figure 3:**
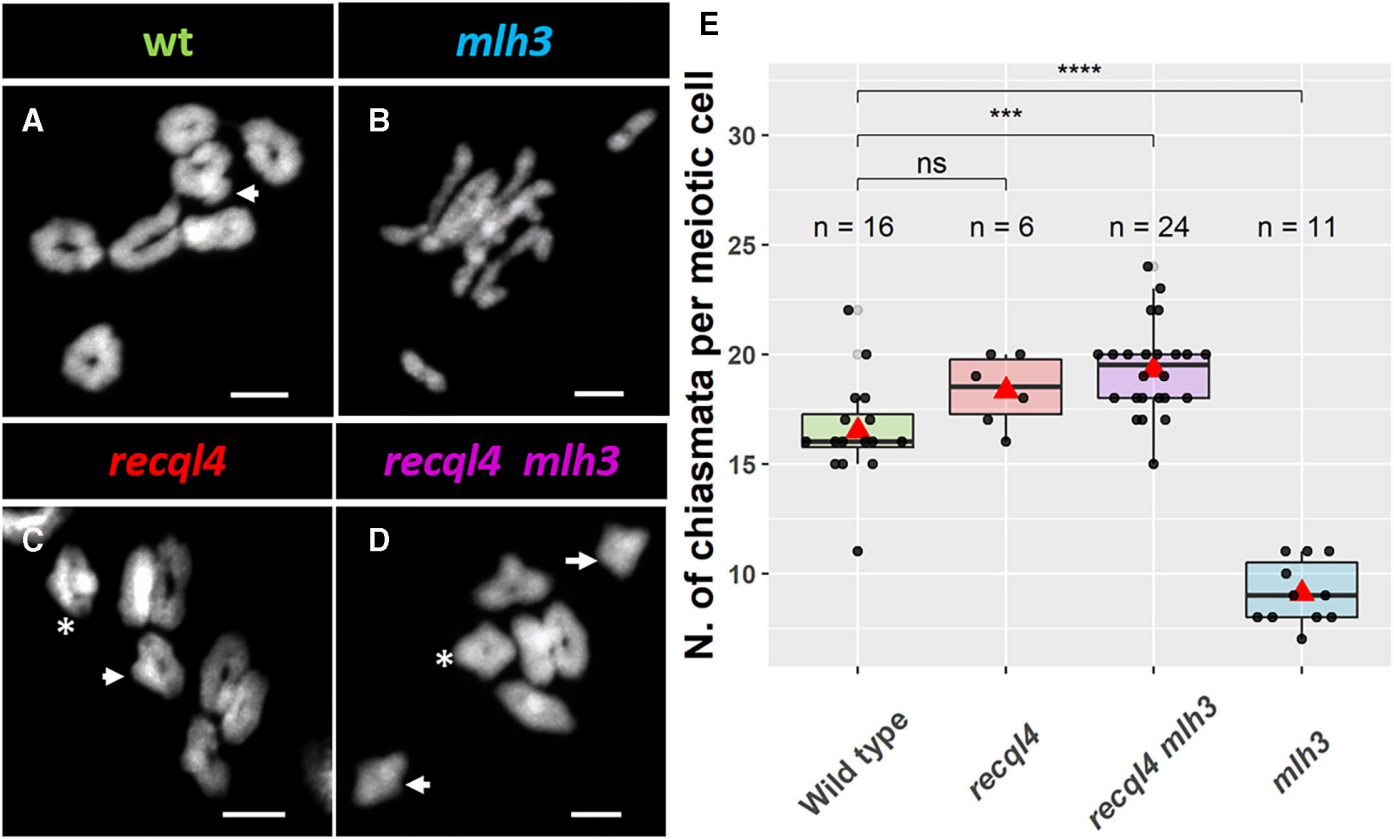
Cytology of male meiosis on F_4_ plants of the different populations: **(A)** WT, **(B)** *mlh3*, **(C)** *recql4*, and **(D)** *recql4 mlh3*. White arrows indicate the bivalents counted with three crossovers and asterisks refer to the bivalents with four crossovers. **(E)** Total chiasmata count per cell and population. The statistical comparison with the WT was carried out with the *t*-test where “ns” is not significant, ***p < 0.001, ****p < 0.0001. The red triangle shows the mean per genotype. Bar size: 10 μm.

Given the WT levels of fertility in the *recql4* and *recql4mlh3* classes, we examined metaphase spreads of the male meiocytes to investigate whether parallel differences could also be detected cytologically. We grew F_4_ plants from randomly selected and genotyped F_3_s that were representative of the four population classes (Figure 3). At metaphase I, the WT population was characterized by the expected seven doughnut-shaped ring bivalents (Figure 3A; Supplementary Figures 5A,E) that relate to the expected presence of two distal chiasmata in the barley chromosomes at meiosis (Higgins et al., 2012; Barakate et al., 2014; Ramsay et al., 2014). Occasional rod bivalents (Supplementary Figure 5E) and ring bivalents with three chiasmata (Figure 3A, marked with an arrow) could also be observed as previously described (Colas et al., 2016) with an average of 16.5 chiasmata per cell (*n* = 16, Figure 3E). In stark contrast, individuals from the *mlh3* population were characterized by a clear abundance of rod bivalents and univalents (Figure 3B), as observed previously on BW230 *des10* (Colas et al., 2016). The reduction of chiasmata compared to WT was significant with an average of 9.1 chiasmata/cell (*n* = 11, Figure 3E). Consistent with the restoration of fertility, both populations containing the *recql4*_G700D_ mutation (Figures 3C,D) showed seven ring bivalents. Some of these appeared to have more than two chiasmata, characterized by the presence of a knob or bump in the ring (Figures 3A,C,D, arrow). Other bivalents, particularly in the double mutant, exhibited a diamond shape (asterisk in Figures 3C,D), which may indicate the presence of multiple chiasmata at the distal ends of a single bivalent. The average number of chiasmata per cell was higher compared to WT with 18.3 (*n* = 6) and 19.3 (*n* = 24) chiasmata/cell for *recql4* and double mutant, respectively, which was due to the presence of the triple and quadruple chiasmata bivalents, though this increase was only significant for the double mutant (Figure 3E). No significant differences were found between the two *recql4* containing families. The results of a more conservative scoring with all the ring bivalents being viewed as representing only two chiasmata are shown in Supplementary Table 2. The scoring of the metaphase spreads was complicated by the “stickiness” observed in the *recql4* containing lines with occasional chromosome bridges being also observed especially in the double mutants (Supplementary Figures 5D,H; Supplementary Figure 6).

### Optimizing Genetic Analysis in the F_3_

**Figure 4:**
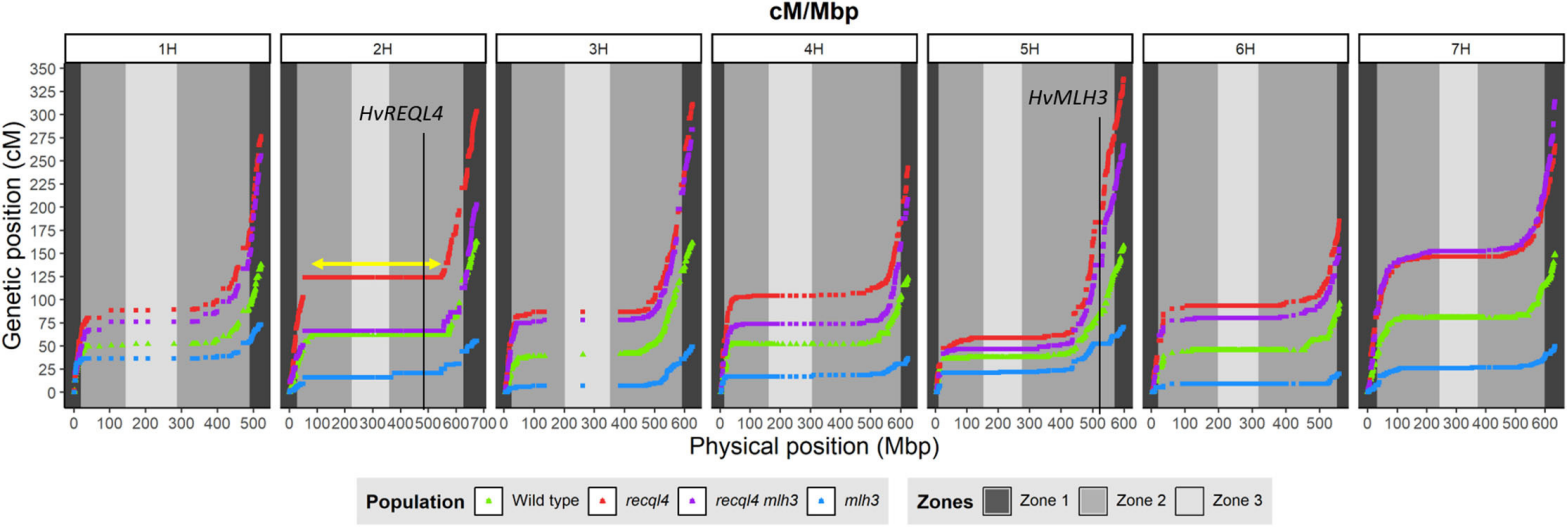
Comparison of the genetic map (cM) and the physical map (Mbp) for each population (WT in green, *mlh3* in blue, *recql4* in red, and the double mutants in purple). The physical positions of *HvRECQL4* and *HvMLH3* are marked with a vertical line in 2H and 5H chromosomes, respectively. The yellow arrow marked in the 2H chromosome indicates the block introgressed together with *HvRECQL4*.

Genetic markers are only informative in F_3_ families if they are heterozygous in the parental F_2_ plants. To optimize the subsequent genetic analysis, heterozygous coverage of the genome was optimized by selecting a combination of the 10 most informative F_2_s from the WT and each *mlh3 recql4* mutant class, and up to 10 F_3_ plants per family were genotyped using the barley 50K SNP array (Bayer et al., 2017). After cleaning the data, 11,965 polymorphic markers remained in population sizes of 95, 86, 93, and 95 individuals for WT, *recql4, mlh3*, and *RECQL4 MLH3* populations, respectively (all the genotyping data is included in Supplementary Material 4). Marker coverage in the distal regions was estimated based on the position of the first and last polymorphic marker for each chromosome on the barley physical map, which was used to determine the percentage of the distal chromosome regions missing from the analysis. Missing coverage in the distal extremities was <0.5% for all the chromosomes, except chromosome 3H, which missed ~0.96% in both the arms, and chromosome 6H, which had 2.74% of missed distal regions, mainly from the long arm (Supplementary Table 3). We then partitioned the markers into the three zones described in Mascher et al. (2017) and in Figure 4. The largest number of the markers was located in interstitial zone 2 followed by the most distal zone 1 though the density of the markers was higher in zone 1. The pericentromeric zone 3 had the lowest number of markers and the lowest density. The number of genetically informative individuals in each population varied along with the genome according to the location of heterozygosity of the parental F_2_s, varying from 10 individuals in the least covered area to 85 individuals in the best. As expected, the number of polymorphic individuals dropped to zero around the position of *MLH3* and *RECQL4*. *RECQL4* was inherited in a large linkage block covering the entire pericentromeric region (part of zone 2 and all of zone 3) of chromosome 2H, while *MLH3* was inherited in a short linkage block in zone 2 of chromosome 5H (gene and introgression are located in Figure 4).

### Assessing Recombination Frequency

**Figure 5:**
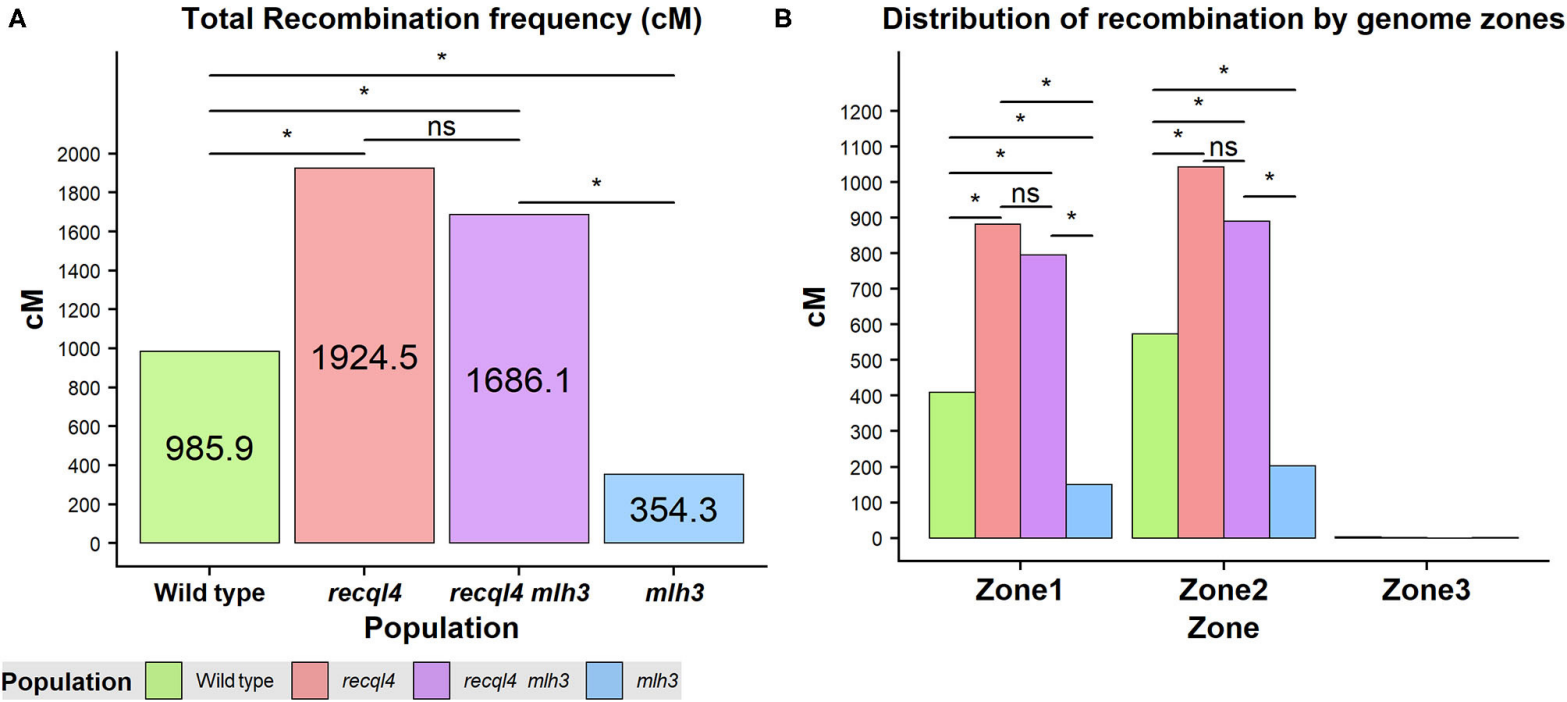
**(A)** Total recombination frequency (cM) for each population. **(B)** Distribution of recombination by the three genomic zones for each population genome-wise. The differences between populations genome wide were tested by the Wilcoxon signed-rank test where *ns* = not significant, *p < 0.05.

Crossover events on the F_2_ generation were estimated as the allele changes between the consecutive markers and were translated into genetic distance (cM) by using the Kosambi mapping function. Both the overall genome wide recombination frequency and total for each chromosome were assessed (Figure 5A; Supplementary Figure 7A). Figure 5 revealed that the population containing only the *recql4*_G700D_ mutation had the highest number of recombination events with a total map length of 1924.5 cM (equivalent to 38.5 COs) followed by the double mutant with 1686.1 cM (33.7 COs), the WT with 985.9 cM (19.7 COs), and finally the *mlh3*_*des*10_ population with 354.3 cM (7.1 COs). This order was maintained for all the chromosomes except for 7H where the double mutant showed the highest recombination (Supplementary Figure 7A). Differences in total recombination between each of the three mutant populations with the WT were significant (the Wilcoxon signed-rank test, Figure 5A). In the case of the *mlh3* population, we observed a significant reduction (*p* < 0.05) in recombination of 63.15% compared to the WT. In contrast, the *recql4* single mutant nearly doubled the WT recombination (95.2% increase, *p* < 0.05), while the increase in the double mutant was slightly less (71% increase, *p* < 0.05) compared to the WT. Comparing the double mutant and *MLH3* populations, indicated an increase of 476% (*p* < 0.05) associated with the presence of *recql4*_G700D_. Finally, in the comparisons between the three mutant populations, significant differences (*p* < 0.05) were found between the two *recql4*_G700D_ containing populations compared with *mlh3*, but not between each other (*p* > 0.05).

### Recombination Distribution

**Figure 6:**
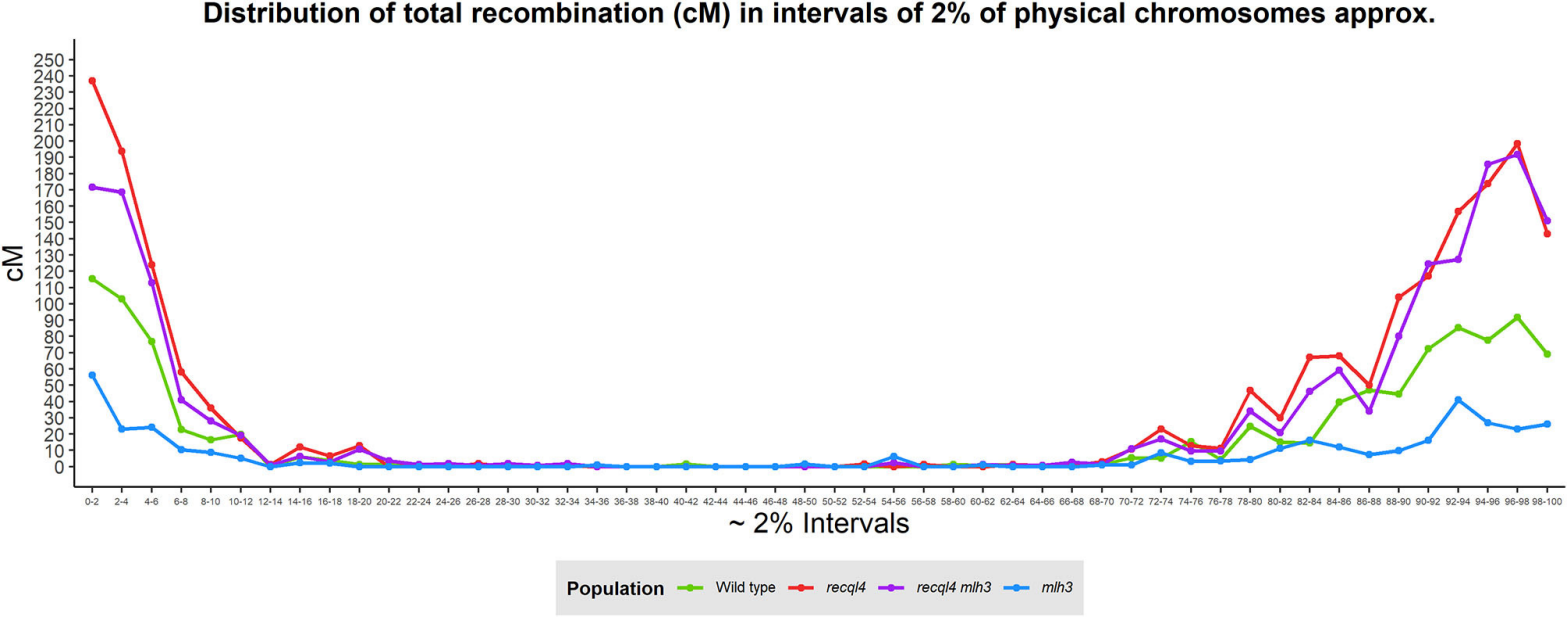
Genome-wide distribution of recombination based on the division of the physical chromosomes in 2% intervals.

Correlations between the genetic (cM) and physical maps (Mbp) (Figure 4) clearly show the well-described skewed patterns of recombination in barley for each chromosome (Mascher et al., 2017). To quantify and compare the distribution of recombination along the chromosomes and between the populations, we plotted recombination frequency in each of the three previously designated genomic zones (Figure 5B) with the short distal zone 1 being gene dense and relatively recombinogenic, the interstitial zone 2 displaying less recombination, and the large pericentromeric zone 3 showing no or very little recombination despite containing a considerable proportion of the gene complement (Mascher et al., 2017). The *recql4* population showed the highest overall levels followed by the double mutant, WT then *mlh3* populations. Chromosome-wise (Supplementary Figure 7B) this order was conserved except for interstitial zone 2 of chromosome 2H where the WT had more recombination compared to double mutant, and chromosome 7H where the double mutant had higher recombination compared to *recql4*_G700D_. The Wilcoxon signed-rank test showed a significant increase of recombination (*p* < 0.05) in both zones 1 and 2 of the *recql4* and double mutant populations and significantly (*p* < 0.05) less recombination in the *mlh3* population compared to the WT. The pericentromeric zone 3 did not show any significant differences in recombination between any of the populations. The difference in the relative distribution of recombination between the zones among the populations was compared by using the contingency table and the chi-squared test (Supplementary Table 4). Interestingly, the WT and *mlh3* populations showed no significant differences between their relative proportions of recombination in zone 1/zone 2, but both the *recql4*_G700D_-containing populations had significantly (*p* < 0.05) more proportional recombination (around 4%) skewed from zone 2 to zone 1. Finally, to provide a higher resolution overview of the distribution of recombination in all the populations, we divided each chromosome into 50 bins, each representing ~2% of the physical length of each chromosome, and combined the amount of recombination (cM) observed in each bin across all seven barley chromosomes into a single measure. Figure 6 shows the resulting recombination landscape for each of the four populations and clearly illustrates the significant increase in both *recql4*_G700D_-containing populations in the first 8% and last 8% of the genome.

## Discussion

In this study, we show that the presence of a single C/T nucleotide change that generates a G700D non-synonymous substitution in *RECQL4* is able to restore fertility to the previously described semi-sterile BW230 *des10* mutant of barley that had shown to contain a 159-bp deletion in *HvMLH3* (Colas et al., 2016). Restoration of fertility in *SuppLine2099* is associated with a restoration of a full-ring bivalent complement at metaphase with a clear indication of additional chiasmata in plants in the *recql4* and *recql4mlh3* classes (Figure 3). Genetic mapping showed that the *recql4* mutation in *SuppLine2099* displayed an unprecedented increase in meiotic recombination frequency to nearly double compared to the WT and over four times higher compared to the *mlh3*_*des*10_ mutant. The increase in recombination associated with mutations in *RECQL4* (*ca*.2.0-fold) is similar to that has been observed in other species, albeit at a slightly lower level. Mieulet et al. (2018) reported that *recql4* driven increases in recombination in a range of species including rice (3.2-fold), pea (4.7-fold), and tomato (2.7-fold), while Serra et al. (2018) and Fernandes et al. (2017) reported an increase of 3.3- and 3.8-fold, respectively, in *Arabidopsis*. However, recombination rates are species-specific and affected by the genomic features (Tiley and Burleigh, 2015) and barley, with a large genome of about 5 Gb, exhibits pronounced, distally skewed recombination (Mayer et al., 2012; Mascher et al., 2017; Schreiber et al., 2020), so it may not be entirely legitimate to compare that had been observed in barley to these other species. Moreover, the comparisons are complicated by the fact that the *RECQL4* mutant studied is potentially not as strong as the exonic T-DNA insertion mutants (Fernandes et al., 2017; Mieulet et al., 2018; Serra et al., 2018) or nonsense-induced TILLING mutants (Mieulet et al., 2018) utilized in the other studies.

The recombination data reported indicates that a non-synonymous G700D amino acid substitution in the conserved helicase domain of *HvRECQL4* results in an increase in recombination exclusively across zones 1 and 2 in the barley genome (Mascher et al., 2017), i.e., the increase occurs in the regions of the barley genome that already undergo recombination in the WT. This is similar to the results for rice shown by Mieulet et al. (2018). The recombination cold pericentromeric zone 3 showed no increase in recombination indicating that the induced increase in recombination frequency was not accompanied by any fundamental change in CO distribution (Mieulet et al., 2018). The relative recombination patterns among the four populations studied in our limited population sizes indicate that in the *recql4*-containing populations, recombination was further skewed toward distal zone 1 with a concomitant reduction in interstitial zone 2. This difference potentially relates to the fundamental constraints on the distribution of recombination in large genome cereals that is driven by the spatial and temporal progression of synapsis from the telomeres to the centromeres and the associated positioning and fate of DSBs and CO intermediates (Higgins et al., 2012).

It is of interest that *recql4*_*G*700*D*_ was identified in our suppressor screen of *BW230 des10*, a genotype that we previously demonstrated contains a deletion in *HvMLH3* (Colas et al., 2016). A mutation in *RECQL4* was previously identified in a suppressor screen in *Arabidopsis* Landsberg erecta by using the class I CO meiotic mutant *msh4* (Séguéla-Arnaud et al., 2015). Intriguingly, suppressor mutations in *RECQL4* were not identified in the earlier screens conducted by using the *Columbia* ecotype due to the gene being duplicated in the *Arabidopsis* genome and *Columbia* containing two functional variants of the gene compared to only one in Landsberg erecta. To the best of our knowledge, a suppressor screen in an *MLH3* mutant background has not been carried out in *Arabidopsis* where the focus has been on the use of ZMM mutants and CO formation with the clearer phenotype that brings. However, it is reassuring that this screen in barley identified a mutant in the helicase domain of *RECQL4* as did the suppressor screen in *msh4* in *Arabidopsis* (Séguéla-Arnaud et al., 2015) highlighting the potential of suppressor screens of non-ZMM mutants to identify the relevant genetic factors and the potential of studies in the non-model species.

Rather than following a traditional and extended positional cloning route to characterize the nature of the mutation in *SuppLine2099*, we used target sequence enrichment of the known meiotic genes combined with the Illumina sequencing to highlight the causal mutation (Schreiber et al., 2019). The restoration of fertility was confirmed in the F_2_ populations (Figure 2) where the percentage of fertile florets in the double *recql4*_*G*700*D*_ *mlh3* mutant plants reached the WT levels. Interestingly, the total number of florets per ear was lower than the WT and *mlh3* in both *recql4*_*G*700*D*_*-*containing mutant populations. Both populations containing the *recql4*_*G*700*D*_ allele inherited the mutation in a large linkage block (approximately sized 450 Mbp) covering the whole of zone 3 and much of zone 2 on chromosome 2H. Given that *cv*. Bowman, the recurrent parent of BW320 *des10* has a reduced number of florets compared to *cv*. Barke (data not shown) and it is possible that the associated changes found in the floret number (Supplementary Figure 4) could be a background effect inherited with the *recql4*_*G*700*D*_ mutation via linkage drag. Alternatively, the phenotype observed could potentially be a direct result of the disrupted function of RECQL4 with an effect on the multiple interactions controlling fertility and inflorescence development in barley. Use of the barley 50K iSelect SNP array allowed robust genome wide genotyping and the length of the WT population genetic map of 985.9 cM is close compared to observed in the previous studies (Li et al., 2010; Zhou et al., 2015; Bayer et al., 2017), despite the reduced mapping power inherent in the use of F_3_ material. It is possible that the recombination observed in our populations may be somewhat underestimated due to marker monomorphism and, in particular, this may explain the lower overall recombination found on chromosome 6H (Supplementary Figure 7A). Nevertheless, the strong reduction of total genetic map length of 63.1% detected in the *mlh3* population in this study was similar though slightly more severe than the 54.1% reported in Colas et al. (2016), though each study used a different cross and the latter used a previous lower density 9K genotyping array.

Metaphase spreads on the *mlh3*_*des*10_mutation-containing F_4_ plants revealed fewer chiasmata compared to the WT with a predominance of rod bivalents (Figure 3) and occasional univalents. This metaphase I phenotype and the comparison to the seven ring bivalents in the WT are entirely congruent with that previously reported for the BW230 *des10* mutant and the WT (Colas et al., 2016). The cytological appearance of bivalents in plants with the *recql4*_*G*700*D*_ mutation was suggestive of multiple distal chiasmata—though detailed counts were impossible to determine. Similar observations have been found previously in the cytological studies in the anti-CO mutants in *Arabidopsis* (Crismani et al., 2012; Séguéla-Arnaud et al., 2015) and reflect the general difficulty in estimating CO numbers from chiasmata counts (Colas et al., 2016). Furthermore, the number of COs estimated from the metaphase spreads is lower compared to those estimated by the genetic maps as observed previously in barley (Ramsay et al., 2014) and these differences are even higher (nearly double) for the *recql4*_*G*700*D*_*-*containing populations, underlining the difficulties of this approach to discern between the closely neighboring chiasmata. The difficulty in assessing CO numbers in the double mutant was potentially exacerbated by the presence of the chromosome bridges at metaphase, which potentially indicates specific issues in the resolution of the repair intermediates.

Both cytological characterization and genetic segregation analysis indicated that the *recql4* single and *RECQL4 MLH3* double mutant had a broadly similar CO phenotype. Both mutants displayed ring bivalents and bivalents with more than two COs with some showing evidence of the chromosome bridges in the double mutant (Supplementary Figure 6). An increase in the average chiasmata/cell counts (Figure 3E) was only found significant for the double mutant (compared to WT), but the number of available cells for the *recql4* population was unfortunately much more limited (*n* = 6). This population was also not significantly different from the double mutant, suggesting the increased trend of *recql4* could be more consistent if more pictures/cells were available. The observations in the double mutant are particularly striking given the severely disrupted phenotype of genotypes containing *mlh3*_*des*10_ alone and are consistent with the genetic data indicating a 4.75-fold increase in recombination in *RECQL4 MLH3*. This level of increase in the recombination mirrors found in the suppressor screens in *Arabidopsis* by using ZMM mutants (Crismani et al., 2012) and corresponds to the restoration of fertility found in *SuppLine2099*. Further studies are needed to confirm whether the increase in CO numbers in the double (and single mutant) is through non-interfering class II COs as reported by Séguéla-Arnaud et al. (2015) and whether the temporal delay in repair events in an *mlh3* background (Colas et al., 2016; Toledo et al., 2019) alters the balance of class I/II CO resulting from the resolution of the repair intermediates.

As shown in the other systems, combining *recql4*_*G*700*D*_ with additional mutations may establish the possibility of inducing even more recombination in barley. Serra et al. (2018) increased recombination levels of a *recql4a recql4b* double mutant of *Arabidopsis* from 3.3- to 3.7-fold by increasing the dosage of the pro-CO E3 ligase *HEI10* via genetic transformation. In the case of anti-CO genes, although it has been reported that they act independently (Séguéla-Arnaud et al., 2015), double mutants do not necessarily result in a further CO increase. Thus, *fancm* on its own induced an increase in recombination in *Arabidopsis*, rice, and pea, but not in the subsequent studies in *Arabidopsis* F_1_ hybrids (Crismani et al., 2012; Mieulet et al., 2018) or wheat (Raz et al., 2021). Moreover, the combination of *fancm* and *recql4* did not show a further increase over *recql4* in *Arabidopsis* or pea (Mieulet et al., 2018). The highest levels of recombination in hybrid *Arabidopsis* were found by combining *recql4* and *figl1* (Fernandes et al., 2017). However, mutating *figl1* induced sterility in rice (Zhang et al., 2017), pea, and tomato (Mieulet et al., 2018). While such undesired and species-specific effects necessitate further study, the use of the mutant alleles in the early stages of a breeding program as suggested by Mieulet et al. (2018) may have the potential to speed up the breeding process.

To conclude, we provide evidence that a mutation in *RECQL4*, as demonstrated in the other species, significantly boosts recombination in a large genome cereal such as barley. The scale of the increase is greater compared to the other approaches that have so far been shown to increase recombination in barley such as temperature (Phillips et al., 2015). The availability of a demonstrated effective non-GM anti-CO allele of *RECQL4* in barley opens the possibility of testing whether increasing recombination will, or will not, have an enabling role in general or specific aspects of crop improvement such as introgression breeding. Given the limits to the plasticity of recombination remain unknown, intriguing options remain to be explored, such as combining *recql4*_*G*700*D*_ in barley with other mutations or combining genetic variants with “treatments” such as temperature (Phillips et al., 2015; Modliszewski et al., 2018), chemicals (Rey et al., 2018), or speed breeding conditions (Ghosh et al., 2018). While we have underlined the potentially important role of *RECQL4* as an anti-CO factor in barley, a major challenge remains in assessing if and how *recql4*_*G*700*D*_ can be used effectively to improve the rate of genetic gain in barley crop improvement.

## Conclusion

By screening for the restoration of fertility of a semi-sterile *HvMLH3* mutant, we identified a non-synonymous suppressor G700D mutation in the anti-CO gene *HvRECQL4*. We show that in both the WT and *Hvmlh3* genetic backgrounds, the G700D mutation increases recombination frequency significantly (ca. 2- and >4-fold, respectively) with the CO distribution showing a stronger skew toward the telomeric regions compared to the WT and no change in the lack of recombination in the pericentromeric regions of the chromosomes. The availability of germplasm containing the *recql4*_*G*700*D*_ mutation will allow us to test the hypothesis that increasing recombination can increase the rate of genetic gain or rapidly reduce the size of introgressed genomic segments from the wild or alien species in the plant breeding programs.

## Data Availability Statement

The original contributions presented in the study are included in the article/Supplementary Materials, further inquiries can be directed to the corresponding author/s.

## Author Contributions

RW and LR conceptualized and supervised the research experiment. MM conducted the experiment to generate the suppressor lines and MS identified the meiotic mutations. MA conducted the experiment work by creating populations to be genotyped and for cytology and drafted the manuscript. Genotype data were processed by MM and PS and analyzed by MA. Cytological samples were prepared by MA and analyzed by IC. LR, RW, IC, MS, and MM reviewed and contributed to improving the manuscript. RW, LR, MA, and IC reviewed last version of the manuscript. All authors contributed to the article and approved the submitted version.

## Funding

This research was funded by the European Community's Seventh Framework Programme FP7/2007–2013 under Grant Agreement No. 222883 MeioSys and by the ERC advanced grant Shuffle (Project ID: 669182). LR and RW were funded from the Scottish Government's Rural and Environment Science and Analytical Services Division Theme 2 Work Program 2.1 and IC was funded by the BBSRC BB/T008636/1.

## Conflict of Interest

The authors declare that the research was conducted in the absence of any commercial or financial relationships that could be construed as a potential conflict of interest.

## Publisher's Note

All claims expressed in this article are solely those of the authors and do not necessarily represent those of their affiliated organizations, or those of the publisher, the editors and the reviewers. Any product that may be evaluated in this article, or claim that may be made by its manufacturer, is not guaranteed or endorsed by the publisher.

## Supplementary Material

Supplementary Material 1 Kompetitive allele-specific PCR (KASP) primer oligos (Excel).

Supplementary Material 2 Parameters in the raw images processed with Imaris (Excel).

Supplementary Material 3 *HvRECQL4* genomic and protein sequence (pdf).

Supplementary Material 4 50K genotyping data (Excel).

Supplementary Figure 1 Suppressor screening population development and process summary.

Supplementary Figure 2 **(A)** Outline of the development of F_3_ populations to be genotyped. **(B)** Selection of the genotypes across the generations to create F_3_ populations. *SuppLine2099* was crossed with *cv*. Barke background to generate heterozygous F_1_s, which were grown to produce F_2_ seed. F_2_ plants were grown and selected by using Kompetitive allele-specific PCR (KASP) genotyping for targeting the segregating mutations. Only the four homozygous combinations were selected to be grown as F_2_ families and produce F_3_ seeds. These were later grown and genotyped with the 50K assay to study the effect of the mutation in F_2_ recombination. The alleles in the mutated genes are indicated by the square box color, being green for the wild type (WT)-like allele, dark red for the *recql4*_*G*700*D*_mutation, and light blue for the *mlh3*_*des*10_ mutation. The gray area in the chromosomes indicates different recombinant backgrounds among the populations.

Supplementary Figure 3 Barchart showing the average fertility of the three plants per selected line in the suppressor screening. *SuppLine2099* was chosen for the present study It is marked in purple and *desynaptic10 (des10)* in blue.

Supplementary Figure 4 Different fertility phenotypes of the selected F_2_ plants. **(A)** Total seeds per ear, **(B)** the Total number of florets per ear, **(C)** Total grams per ear, **(D)** Thousand grain weight (TGW). The statistical comparisons were done with the *t*-test where “ns” is not significant, ^*^*p* < 0.05, ^**^*p* < 0.01, ^***^*P* < 0.001, ^****^*p* < 0.0001. All the comparisons are done by using the WT as a reference. The red triangle shows the mean per genotype. Each dot in the graph represents the average of 3 years of one plant, except for grams per year and TGW, where all the ears of the plants were used.

Supplementary Figure 5 Cytology of male meiosis on the F_4_ plants of different populations: **(A,E)** WT, **(B,F)** *mlh3*, **(C,G)** *recql4*, and **(D,H)** *recql4mlh3*. Chiasmata counts are pointed with the gray dots (when discernible) and the chromosome bridges marked with white arrows. Bar size: 5 μm.

Supplementary Figure 6 Quantitative scoring of the chromosome bridges per meiotic cell. The statistical comparisons are done with the *t*-test where “ns” is not significant, ^****^*p* < 0.0001. All the comparisons are done by using the WT as a reference.

Supplementary Figure 7 **(A)** Genetic chromosome length (cM) of every chromosome for each population. **(B)** Distribution of recombination by the genomic zones for each population chromosome-wise.

Supplementary Table 1 Exome capture output and mutation details of the lines selected in the suppressor screening. *SuppLine2099* (first row) was selected for this study.

Supplementary Table 2 Rescoring of chiasmata counts by using a more conservative approach of counting only the ring bivalents.

Supplementary Table 3 Polymorphic marker summary. Details of distal regions not covered by polymorphic markers.

Supplementary Table 4 Relative percentage of recombination contained in each genomic zone for each population. The significance is calculated by the contingency table and the chi-squared test of the proportions of recombination in each population compared to the WT control (ns = not significant, ^*^*p* < 0.05, ^**^*p* < 0.001).

## References

[B1] Barakate A. Higgins J. D. Vivera S. Stephens J. Perry R. M. Ramsay L.. (2014). The synaptonemal complex protein ZYP1 is required for imposition of meiotic crossovers in barley. Plant Cell 26, 729–740. 10.1105/tpc.113.121269 24563202 PMC3967036

[B2] Bayer M. M. Rapazote-Flores P. Ganal M. Hedley P. E. Macaulay M. Plieske J.. (2017). Development and evaluation of a barley 50k iSelect SNP array. Front. Plant Sci. 8:1792. 10.3389/fpls.2017.01792 29089957 PMC5651081

[B3] Berchowitz L. E. Copenhaver G. P. (2010). Genetic interference: don't stand so close to me. Curr. Genomics 11, 91–102. 10.2174/138920210790886835 20885817 PMC2874225

[B4] Blary A. Jenczewski E. (2019). Manipulation of crossover frequency and distribution for plant breeding. Theor. Appl. Genet. 132, 575–592. 10.1007/s00122-018-3240-1 30483818 PMC6439139

[B5] Caldwell D. G. McCallum N. Shaw P. Muehlbauer G. J. Marshall D. F. Waugh R. (2004). A structured mutant population for forward and reverse genetics in Barley (*Hordeum vulgare* L.). Plant J. 40, 143–150. 10.1111/j.1365-313X.2004.02190.x 15361148

[B6] Choi Y. Sims G. E. Murphy S. Miller J. R. Chan A. P. (2012). Predicting the functional effect of amino acid substitutions and indels. PLoS ONE 7:e46688. 10.1371/journal.pone.0046688 23056405 PMC3466303

[B7] Colas I. Macaulay M. Higgins J. D. Phillips D. Barakate A. Posch M.. (2016). A spontaneous mutation in MutL-Homolog 3 (HvMLH3) affects synapsis and crossover resolution in the barley desynaptic mutant des10. New Phytol. 212, 693–707. 10.1111/nph.14061 27392293

[B8] Copenhaver G. P. (2003). Crossover interference in *Arabidopsis*. Am. J. Hum. Genet. 73, 188–197. 10.1086/376610 12772089 PMC1180580

[B9] Crismani W. Girard C. Froger N. Pradillo M. Santos J. L. Chelysheva L.. (2012). FANCM limits meiotic crossovers. Science 336, 1588–1590. 10.1126/science.1220381 22723424

[B10] Darrier B. Rimbert H. Balfourier F. Pingault L. Josselin A. A. Servin B.. (2017). High-resolution mapping of crossover events in the hexaploid wheat genome suggests a universal recombination mechanism. Genetics 206, 1373–1388. 10.1534/genetics.116.196014 28533438 PMC5500137

[B11] Devaux P. Kilian A. Kleinhofs A. (1995). Comparative mapping of the barley genome with male and female recombination-derived, doubled haploid populations. Mol. Gen. Genet. 249, 600–608. 10.1007/BF00418029 8544825

[B12] Druka A. Potokina E. Luo Z. Jiang N. Chen X. Kearsey M.. (2010). Expression quantitative trait locianalysis in plants. Plant Biotechnol. J. 8, 10–27. 10.1111/j.1467-7652.2009.00460.x 20055957

[B13] Fernandes J. B. Duhamel M. Seguéla-Arnaud M. Froger N. Girard C. Choinard S.. (2018). FIGL1 and its novel partner FLIP form a conserved complex that regulates homologous recombination. PLoS Genet. 14:e1007317. 10.1371/journal.pgen.1007317 29608566 PMC5897033

[B14] Fernandes J. B. Seguéla-Arnaud M. Larchevêque C. Lloyd A. H. Mercier R. (2017). Unleashing meiotic crossovers in hybrid plants. Proc. Natl. Acad. Sci. U.S.A. 2017:13078. 10.1101/159640 29183972 PMC5877974

[B15] Ghosh S. Ramirez-Gonzalez R. H. Simmonds J. Wells R. Rayner T. Hafeez A.. (2018). Speed breeding in growth chambers and glasshouses for crop breeding and model plant research. Nat. Protoc. 13, 2944–2963. 10.1038/s41596-018-0072-z 30446746

[B16] Girard C. Chelysheva L. Choinard S. Froger N. Macaisne N. Lehmemdi A.. (2015). AAA-ATPase FIDGETIN-LIKE 1 and helicase FANCM antagonize meiotic crossovers by distinct mechanisms. PLoS Genet. 11:e1005369. 10.1371/journal.pgen.1005369 26161528 PMC4498898

[B17] Higgins J. D. Buckling E. F. Franklin F. C. H. Jones G. H. (2008). Expression and functional analysis of *AtMUS81* in *Arabidopsis* meiosis reveals a role in the second pathway of crossing-over. Plant J. 54, 152–162. 10.1111/j.1365-313X.2008.03403.x 18182028

[B18] Higgins J. D. Perry R. M. Barakate A. Ramsay L. Waugh R. Halpin C.. (2012). Spatiotemporal asymmetry of the meiotic program underlies the predominantly distal distribution of meiotic crossovers in barley. Plant Cell 24, 4096–4109. 10.1105/tpc.112.102483 23104831 PMC3517238

[B19] Jayakodi M. Padmarasu S. Haberer G. Bonthala V. S. Gundlach H. Monat C.. (2020). The barley pan-genome reveals the hidden legacy of mutation breeding. Nature 588, 284–289. 10.1038/s41586-020-2947-8 33239781 PMC7759462

[B20] Li H. Kilian A. Zhou M. Wenzl P. Huttner E. Mendham N.. (2010). Construction of a high-density composite map and comparative mapping of segregation distortion regions in barley. Mol. Genet. Genomics 284, 319–331. 10.1007/s00438-010-0570-3 20803217

[B21] Lundqvist U. Franckowiak J. Konishi T. (1997). New and revised descriptions of barley genes. Barley Genet. Newsl. Med. Sci. Hist. Soc. 26. 22–516.

[B22] Mascher M. Gundlach H. Himmelbach A. Beier S. Twardziok S. O. Wicker T.. (2017). A chromosome conformation capture ordered sequence of the barley genome. Nature 544, 427–433. 10.1038/nature22043 28447635

[B23] Mayer K. F. X. Waugh R. Langridge P. Close T. J. Wise R. P. Graner A.. (2012). A physical, genetic and functional sequence assembly of the barley genome. Nature 491, 711–716. 10.1038/nature11543 23075845

[B24] Mercier R. Jolivet S. Vezon D. Huppe E. Chelysheva L. Giovanni M.. (2005). Two meiotic crossover classes cohabit in *Arabidopsis*: One is dependent on *MER3*, whereas the other one is not. Curr. Biol. 15, 692–701. 10.1016/j.cub.2005.02.056 15854901

[B25] Mercier R. Mézard C. Jenczewski E. Macaisne N. Grelon M. (2015). The molecular biology of meiosis in plants. Annu. Rev. Plant Biol. 66, 297–327. 10.1146/annurev-arplant-050213-035923 25494464

[B26] Mieulet D. Aubert G. Bres C. Klein A. Droc G. Vieille E.. (2018). Unleashing meiotic crossovers in crops. Nat. Plants 4, 1010–1016. 10.1038/s41477-018-0311-x 30478361

[B27] Modliszewski J. L. Wang H. Albright A. R. Lewis S. M. Bennett A. R. Huang J.. (2018). Elevated temperature increases meiotic crossover frequency via the interfering (Type I) pathway in *Arabidopsis thaliana*. PLoS Genet. 14:e1007384. 10.1371/journal.pgen.1007384 29771908 PMC5976207

[B28] Monat C. Padmarasu S. Lux T. Wicker T. Gundlach H. Himmelbach A.. (2019). TRITEX: chromosome-scale sequence assembly of Triticeae genomes with open-source tools. Genome Biol. 20:284. 10.1186/s13059-019-1899-5 31849336 PMC6918601

[B29] Phillips D. Jenkins G. Macaulay M. Nibau C. Wnetrzak J. Fallding D.. (2015). The effect of temperature on the male and female recombination landscape of barley. New Phytol. 208, 421–429. 10.1111/nph.13548 26255865

[B30] Ramsay L. Colas I. Waugh R. (2014). “Modulation of meiotic recombination,” in Biotechnological Approaches to Barley Improvement, eds J. Kumlehn and N. Stein (Berlin; Heidelberg: Springer), 311–329. 10.1007/978-3-662-44406-1_16

[B31] Raz A. Dahan-Meir T. Melamed-Bessudo C. Leshkowitz D. Levy A. A. (2021). Redistribution of meiotic crossovers along wheat chromosomes by virus-induced gene silencing. Front. Plant Sci. 11:635139. 10.3389/fpls.2020.635139 33613593 PMC7890124

[B32] Rey M. Martín A. C. Smedley M. Hayta S. Harwood W. Shaw P.. (2018). Magnesium increases homoeologous crossover frequency in *ZIP4* (*Ph1*) mutant wheat-wild relative hybrids. Front. Plant Sci. 9:509. 10.1101/278341 29731763 PMC5920029

[B33] Schreiber M. Barakate A. Uzrek N. Macaulay M. Sourdille A. Morris J.. (2019). A highly mutagenised barley (*cv*. Golden Promise) TILLING population coupled with strategies for screening - by - sequencing. Plant Methods 15:99. 10.1186/s13007-019-0486-9 31462905 PMC6708184

[B34] Schreiber M. Mascher M. Wright J. Padmarasu S. Himmelbach A. Heavens D.. (2020). A genome assembly of the barley ‘transformation reference' cultivar golden promise. G3 Genes Genomes Genet. 10, 1823–1827. 10.1101/2020.02.12.945550 32241919 PMC7263683

[B35] Séguéla-Arnaud M. Crismani W. Larchevêque C. Mazel J. Froger N. Choinard S.. (2015). Multiple mechanisms limit meiotic crossovers: TOP3α and two BLM homologs antagonize crossovers in parallel to FANCM. Proc. Natl. Acad. Sci. U.S.A. 112, 4713–4718. 10.1073/pnas.1423107112 25825745 PMC4403193

[B36] Serra H. Lambing C. Griffin C. H. Topp S. D. Nageswaran D. C. Underwood C. J.. (2018). Massive crossover elevation via combination of *HEI10* and *recq4a recq4b* during *Arabidopsis* meiosis. Proc. Natl. Acad. Sci. U.S.A. 115, 2437–2442. 10.1073/pnas.1713071115 29463699 PMC5877939

[B37] Tiley G. P. Burleigh J. G. (2015). The relationship of recombination rate, genome structure, and patterns of molecular evolution across angiosperms. BMC Evol. Biol. 15:194. 10.1186/s12862-015-0473-3 26377000 PMC4574184

[B38] Toledo M. Sun X. Brieño-Enríquez M. A. Raghavan V. Gray S. Pea J.. (2019). A mutation in the endonuclease domain of mouse MLH3 reveals novel roles for MutLγ during crossover formation in meiotic prophase I. PLoS Genet. 15:e1008177. 10.1371/journal.pgen.1008177 31170160 PMC6588253

[B39] Wang Y. Copenhaver G. P. (2018). Meiotic recombination : mixing it up in plants. Annu. Rev. Plant Biol. 69, 577–609. 10.1146/annurev-arplant-042817-040431 29489392

[B40] Wijnker E. de Jong H. (2008). Managing meiotic recombination in plant breeding. Trends Plant Sci. 13, 640–646. 10.1016/j.tplants.2008.09.004 18948054

[B41] Zhang P. Zhang Y. Sun L. Sinumporn S. Yang Z. (2017). The Rice AAA-ATPase OsFIGNL1 is essential for male meiosis. Front. Plant Sci. 8:1639. 10.3389/fpls.2017.01639 29021797 PMC5624289

[B42] Zhou G. Zhang Q. Zhang X. Tan C. Li C. (2015). Construction of high-density genetic map in barley through restriction-site associated DNA sequencing. PLoS ONE 10:e0133161. 10.1371/journal.pone.0133161 26182149 PMC4504713

