# Supplementary material for: An Induced Mutation in HvRECQL4 Increases the Overall Recombination and Restores Fertility in a Barley HvMLH3 Mutant Background
Source: Front Plant Sci. 2021 Nov 12;12:706560. doi: 10.3389/fpls.2021.706560 (PMC8633572; doi:10.3389/fpls.2021.706560)
Supplement: Supplementary Material 3 — HvRECQL4 genomic and protein sequence (pdf). [file Data_Sheet_3.pdf]

>RecQl4
ATGCAAGGAAGCAACAAGCTGAATGCTGGCTCCAACTGCAACGACAAGCTGCCAAAAGTCAACTG
GCCACATCATGCAAATGCTATACAAAGCTCCCTTAGCAAAGGTGACTTTCTGAGTTCAAGCTTTCT
GTTCTCTTTACCAACACAAAGGGCAAATCCAGAAGCAAACTGCAATACAATGCATTCTTTAAGGTC
CGCTGCTTGTAAAATTCAAGGCCCAGAACGTCTTCAAGTTCCATGGATCGAGAAGGCCTGGCGTTC
TGTGTGTAACACCCAGGTGACCTGTAAGAATTATTTAAGACCTGGTTTATCTGCAAAAGTGAAAGA
TTGTGGCAGGGATTTTGCTCTTACTTATGCAGCAGATTCTTCATACAATGCCAAAAAACTGGATAA
TGTGCCGAAAAGTACAATTCTCTCTGAGGGAAGTCTGCATCAACGCACTGAAAGTGGTATTCTGGA
GCAAAATAGCAGTCATCGGACCTGTACGATGGCTTACCAGAGCAATCATGTGGTTGGAACAACAT
ATCAGAGCAGTTTCGCCAGAACTGATGCTATGTCATGCCAGACTGTTCCTGTTGCTGATAACATGT
GTGCTGATGATAAGTTAGATGCCATGGATGATGACGAGATTCTGGCGAGTATTGACGTGGACCGC
ATAGTTATGGAACATTATGAAGCAACAAATACACCCAGAGGGTTAGCATCCCGACAAATGTCAAC
TCCATCAGGAAACAAGTTTAACTTCACAGGTTTAGACGAGAACAGTTTACCACAAGAACTCTCTGA
AATTTGCAGTCATGGTTACAAGTTAGCTTTTTGCCCAGAGGCGAATTATCATTTGCAAGGGATGAA
GGATCAGTTGATTTCAGTATCCAATAAACTTCTTGATGGTTCTGGGGAACTCAGTCCTCAACATTCT
GAAGAGCTTCGTCAACAGCGTGCACATCTAAATAAACAGATTCAGATACTTGGGGATTATATGGC
GAGGCCAACCCAAGACGATGAGCGGAAGAGATCACACTCTATGGCCTCCACAACAGCTGCAGAAG
GGCATCGACCCCCTATGACCCCAAGCACTTTTGTGGACAATAATAGATCCCAATCTCAGTTTTATG
ATACGAATGGACCCTGGGACGGTGGCTCATGCTACACTCCTGCTCCATGTCCCTACATGGATAGTA
ACATACCGTTAACATCGGTACAGAGAGATTACACCCGTAGAAATATAGATATTAGCTACACTGAT
GGTTCTGGTGATAAGAAGTGGAGCAGCACAGATTTTCCATGGACTAAGGAACTTGAGGTCCACAA
CAAGAGAGTATTTGGAAACCGTTCTTTCCGCCCAAATCAGCGAGAAATAATTAATGCCACAATGA
ATGGGAGTGATGTTTTTGTTTTGATGCCAACTGGTGGTGGAAAGAGTTTGACCTACCAGCTTCCAG
CTCTCATTGAGGAGGGCATAACGCTAGTAGTTTGTCCTCTTGTTTCACTCATCCAGGACCAGATCAT
GCATTTATCGCAGGCAAATATCCCTGCAACTTATCTCAGTGCCAATTTGGAATGGACAGAACAGCA
GAGAATATTAAGAGATCTAATGTCTCCTACATCTACATGTAACTATAAGTTACTGTATGTTACTCC
AGAAAAAATAGCTAAGAGTGATGTTCTACTGAGACAATTGGAAAATTTATATTCACGAGGTTATCT
TTCTAGAATTGTTATTGATGAAGCTCATTGTGTAAGCCAGTGGGGACATGATTTCCGACCTGATTA
CCAGCATTTAGGTGTCTTGAAACAAAAGTTTCCAGAGACACCAGTCCTGGCCTTGACTGCAACAGC
AACTGCGAGTGTAAAGGAAGATGTTGTGCAAGCTCTAGGCCTAGCAAACTGTGTTGTTTTCAAACA
GAGTTTTAATCGTCCCAATCTAAGGTATTTTGTGATGCCCAAGACAAAGAAATGCCTCGAGGATAT
AGATCGCTTTATCCGTGAAAATCATCATAAAGAATGTGGCATCATATATTGCCTTTCAAGAATGGA
CTGTGAAAAAGTGGCTGAAAAACTGAGGGAATATGGGCACCAAGCATCACACTATCATG[G]TAAC
ATGGAGCCTTTTGATAGAACACAAGTTCAGAGGCTGTGGAGTATGGATAAGATAAACATAATCTG
TGCTACAGTTGCATTTGGGATGGGTATCAATAAACCCGATGTCCGTTTTGTTATTCATCATTCCCTT
CCCAAATCAATTGAAGGGTACCATCAGGAGTGTGGACGTGCTGGCAGAGATGGACAACGCTCATC
TTGTGTGCTGTATTATAACTATTCTGACTATATTCGTGTCAAACACATGATTACACAAGGATCTGCA
GAGCAAGTAAGATCAAGCTCTTCGTCTTCACATGGGCAAGCACTTGCAACACACAAGGAGAATCT
CTTGTGCATGGTTAGTTACTGTGAAAATGATGTGGATTGCAGACGTTTACTTCAACTGATACACTTC
GGAGAGACTTTTGATCCATCACATTGTGCAAAAACATGTGATAATTGCAAAAAAGGATTGAGATG
GATTGAAAAAGATGTGACCAATATTGCCAAGCAATTGGTCGAGCTGGTCTTGGCGACAGGGCAGC
CATGTTCAAGTTCTCATATTCTTGAAGTTTACAGGGGTTCCTTGAACCAAAATGTCAAGAAGAACC
GTCATGATATGTTGCCTCTTCATGGAGCTGGAAAGCATCTAGCTAAAGGTGAGGCAGCAAGAGTA
CTACGGAATCTAGTTACTGAGGGAATACTTGCTGAGGATGTCAAAAAGAGCGATACATATGGATC
AGTATCATCTGTTTTAAAGGTGAATCAGGTTAAAGTTGGTGGTCTTCGCTCTGGCAATCAGATCGT
CCTTAAGTTTCCCACTCCTGACAAGGCCCCTAAGATGGGGAAACTTGACGAATCATCAATCTCACA
AGTCAATAAGCCTGTTCAACGGCAGAGTGAAATGGATGAGAATTTTTCATCGTTGCTCTATGAAAC
TTTAAGAATCCTTAGGTCTCAGATAGCAGAGGCTGCAGGATGTGGTGTACACCACATATTTAATAA
TGAAACACTGAAGGAAATTAGCACTCGGGTACCAAGGACAAAAGAAGAACTTTTGGAGATAAAC
GGCATCGGCAAGGTGAAGCTCAACAAGTATGGAGATCGCGTACTTGCAACCTTAGAGGAGTTTCT
CAACCAATTTTCAAGCGGAAGCAAGAGAAGCAGCAGCAGCGGTGGCAGCAACGAGCAAAACGAG
GCCGTGAAGAAGCGAAGGGGCTTCACCGCCATCGACACCTCTGGGAACGGCAATGACTTTGAAGA
ACGCACAGTTCAGTCCAAGAAACACGCAGGAAAGACACAAAACAGCAAGCAGGGAATAGCCGAT
GCTGCAAGCGTGATCCAAGACATCCGCTATATAGATGTTGACTTGGACGGATGCGAAGATGTGGA
TGAAGAGCTGTGCAGCAGTGTCCAACAGCCCGTGGCCTCTGGTAGGGTTTTGCCCAAGTGGTCAGC
CGGCGGTAATGCCCCCCCACCTAATATATTTGAAGAATTTAAATACACCAACTAG

>RecQl4Genomic
CACCTCAGTTTGGTTTTGGCTTCTGATTTTGTGAGATCTTTATCACTCAACATTCAATAGCACTTCA
TGCCCTAATTATAATAGCAGCGA
ATGCAAGGAAGCAACAAGCTGAATGCTGGCTCCAACTGCAACGACAAGCTGCCAAAAGTCAACTG
GCCACATCATGCAAATGCTATACAAAGCTCCCTTAGCAAAGGTGACTTTCTGAGTTCAAGCTTTCT
GTTCTCTTTACCAACACAAAGGGCAAATCCAGAAGCAAACTGCAATACAATGCATTCTTTAAGgtata
cttctgttgtatgcaattagttgatcttatcctagaaacagctgttcattgcatcagcaacaaaactcatattcccagGTCCGCTGCTTGTAAAATTCA
AGGCCCAGAACGTCTTCAAGTTCCATGGATCGAGAAGgtatggcaataatactgtaacatgctatgaatcatcacacaatgctta
aaaatgctatagctacacgatcacttcaacctgtagattttgctaatcttaatgtaagaagcagttttctttcaaaaccatattataaaagctattggatgcttgtttgtcttga
ctaagtgcaaaactaggacagtttgttaagaacttgttagctgtctactaaaggcgtatgacatttttttgggagttaggactggttacctgcttgctgcatgcctgtaagtt
caatcctggcttgtcttttacttttagtcatcactntgcctctgaagatcagtgtgttctgttcacttttgtgtggttagtggtatcctgcatgcctgtaaatttaatcctggctt
gtcttttacttccagtgtatcgctttgctgaaaaaaaaagcgtaaacctaattgccactacctaaaatgtccagtgtgctctgttcgtttttgtgtggttgttagtattctaatg
aaacttctatgacagGCCTGGCGTTCTGTGTGTAACACCCAGGTGACCTGTAAGAATTATTTAAGACCTGGT
TTATCTGCAAAAGTGAAAGATTGTGGCAGGGATTTTGCTCTTACTTATGCAGCAGATTCTTCATAC
AATGCCAAAAAACTGGATAATGTGCCGAAAAGTACAATTCTCTCTGAGGGAAGTCTGCATCAACG
CACTGAAAGTGGTATTCTGGAGCAAAATAGCAGTCATCGGACCTGTACGATGGCTTACCAGAGCA
ATCATGTGGTTGGAACAACATATCAGAGCAGTTTCGCCAGAACTGATGCTATGTCATGCCAGACTG
TTCCTGTTGCTGATAACATGTGTGCTGATGATAAGTTAGATGCCATGGATGATGACGAGATTCTGG
CGgtaggtgcttccatcccataaatttgatttgatattttatctaccgaattatcttcccagacttagatgaacataccatattatcatgtcataattggtgacaaatctagtt
atgttccagAGTATTGACGTGGACCGCATAGTTATGGAACATTATGAAGCAACAAATACACCCAGAGGG
TTAGCATCCCGACAAATGTCAACTCCATCAGGAAACAAGTTTAACTTCACAGGTTTAGACGAGAAC
AGTTTACCACAAGAACTCTCTGAAATTTGCAGTCATGGTTACAAGgtaagcaaagtattagttgaatcatcattcatgctg
tttaggtagtagctatttcagcatgttagctcacaagttaatacaaaacttgtgcagTTAGCTTTTTGCCCAGAGGCGAATTATCATTTG
CAAGGGATGAAGGATCAGTTGATTTCAGTATCCAATAAACTTCTTGATGGTTCTGGGGAACTCAGT
CCTCAACATTCTGAAGAGCTTCGTCAACAGCGgtttggttcttgggcctactcattatttttgtgtatattatgaagcttgatttaccagtatt
aatactgatattttctgtggttatacactgcagTGCACATCTAAATAAACAGATTCAGATACTTGGGGATTATATGGCGA
GGCCAACCCAAGACGATGAGCGGAAGAGATCACACTCTATGGCCTCCACAACAGCTGCAGAAGGG
CATCGACCCCCTATGACCCCAAGCACTTTTGTGGACAATAATAGATCCCAATCTCAGTTTTATGAT
ACGAATGGACCCTGGGACGGTGGCTCATGCTACACTCCTGCTCCATGTCCCTACATGGATAGTAAC
ATACCGTTAACATCGGTACAGAGAGATTACACCCGTAGAAATATAGATATTAGCTACACTGATGGT
TCTGGTGATAAGAAGTGGAGCAGCACAGATTTTCCATGGACTAAGGAACTTGAGgttatttattttcttcaaaatc
aatagttggttacttcatcaacattggctcatgagaatcactaattttgagtgcatcttcagGTCCACAACAAGAGAGTATTTGGAAACCG
TTCTTTCCGCCCAAATCAGCGAGAAATAATTAATGCCACAATGAATGGGAGTGATGTTTTTGTTTT
GATGCCAACTGGTGGTGGAAAGAGTTTGACCTACCAGgtaataaaccatgcttctacaattttccatccttgtttgatatgatatatg
catctttgaaaattctactgtatacaccttttgactcttcagtggtgatttttgcgaatttgttactcctttgcctttgtcttttctagtgctcacatatatatgatgctactgacca
agtggtacttaagttttaaacatggaagaggagataatttcttaagagtttgatcagatgagagttatgaattaatcaatggtcttgaaggaaattaaaacaaaccaattt
attcagtattgactgctaactccatctgcaaggttttatttcctcaaagtagcactgaacacttgtctattaatttgcagCTTCCAGCTCTCATTGAGGA
GGGCATAACGCTAGTAGTTTGTCCTCTTGTTTCACTCATCCAGGACCAGATCATGCATTTATCGCAG
gtaatacattatctcatttcctataacatgattcttgatgctttttttgaccactcacctcctgtctggagcagGCAAATATCCCTGCAACTTATCTC
AGTGCCAATTTGGAATGGACAGAACAGCAGAGAATATTAAGAGATCTAATGTCTCCTACATCTAC
ATGTAACTATAAGTTACTGTATGTTACTCCAGAAAAAATAGCTAAgtaagtggaatgtcttaaattccacatgacaggtc
tgaagtagttagctgcatcatctcttaaccgctgtttccaaattatcctttttgcaatatagtgtaacctccatacttacttcataggtggcattgtttagtagttcaccatttcc
agacaaaattagctttcacagttcctttttttacctaacatctacatttgaaatccctgaatgcacttaaggcttaagctctctattagtccctcaccttgtacagttatactctt
ctctgttagtatatcatgtactccctccgttcctaaatataagaccttttagagattccactatgaactacatacggatgtatatagacatactttagagtgtaggttcactca
ttttactccgtatgtagtctatagtgaaatctctaaaaggtcttatatttaggaacagagggagtaggtctgttttactatcaatgcatttggtagtaactggtagcaatttcc
tgggtacagGAGTGATGTTCTACTGAGACAATTGGAAAATTTATATTCACGAGGTTATCTTTCTAGAATT
GTTATTGATGAAGCTCATTGTGTAAGCCAGTGGGGACATGATTTCCGACCTGATTACCAGgtactttttatc
aggaagatatatgttcatgtcaaataagagacacccatttgatatagcttttgtacatgatctcagCATTTAGGTGTCTTGAAACAAAAGTTT
CCAGAGACACCAGTCCTGGCCTTGACTGCAACAGCAACTGCGAGTGTAAAGGAAGATGTTGTGCA
AGCTCTAGGCCTAGCAAACTGTGTTGTTTTCAAACAGAGTTTTAATCGTCCCAATCTAAGgtaagtctgtt
agaaaattttgacgtacaacttctattgcacaaaccatctcactgcaagttttgcagGTATTTTGTGATGCCCAAGACAAAGAAATGCC
TCGAGGATATAGATCGCTTTATCCGTGAAAATCATCATAAAGAATGTGGCATCATATATTGCCTTT
CAAGAATGGACTGTGAAAAAGTGGCTGAAAAACTGAGGgttacttcttcagactttaagctgttcactcttcacgcatgctgtgtt
ctctttcaatatttagtattgtttgctggagcaaattcgtcctaaccaatcttttcagtattctcatgtgttttccaccttttgctgaatatagatcttcaattttattttgagtcaat
cctcttgtttatcctgattcctgagtgcctgtctataggtaaacgtctttaccactacggaatatagaggaacacacgtcagtgaaaaagggctatatattttatgtgttatt
ggggccccatagttgtaaaggagtcacactttggttttaggctccgtttggtaaaattagttacacacgttggtttgcaccatcaacaattcctacctgacttacgtttcat
gtgtaaactaataatctaatgtagatgcaccaaatcttagaattctgtttagatgtttcgaaaggtaatacacagccatgttgaatatagttggttccttaactcaaatgtgc
cggatagagggtttggtagtgcacagctcatgatatttttgcccagatttactacatgtcgcagtctcattacatgcctaagtgggggccatgtcaggacctgatggttt
tagagtgcataagttgatgtcaacattttaattgtgctagtcggatgatgtgataaatcccaaggctgtactcggatttactcagaaaatagaaatctgatgggctatgca
aatttgaagcgcttagcatttgaactactgacccgcctgacatttttttgtccgtttcctgtttattgacacagGAATATGGGCACCAAGCATCACA

CTATCATG[G]TAACATGGAGCCTTTTGATAGAACACAAGTTCAGAGGCTGTGGAGTATGGATAAG
ATAAACATAATCTGTGCTACAGTTGCATTTGGGATGGgtaatgtgatctttcaaacatcttcaaaagaaatatttatctgtgctccat
gttaatgttccgatttgcagGTATCAATAAACCCGATGTCCGTTTTGTTATTCATCATTCCCTTCCCAAATCAATT
GAAGGGTACCATCAGgtaagagaatgtttgcacaggaaactgcatgatttaagctgtttcatgaccgagttttcttttagGAGTGTGGACGT
GCTGGCAGAGATGGACAACGCTCATCTTGTGTGCTGTATTATAACTATTCTGACTATgtaagtttagtggtac
cttgattgacatatcttgtcagcctgtcatctcattggtcatcacaaacaagtaattttcttgtcaaactatctagATTCGTGTCAAACACATGATTA
CACAAGGATCTGCAGAGCAAGTAAGATCAAGCTCTTCGTCTTCACATGGGCAAGCACTTGCAACA
CACAAGGAGAATCTCTTGTGCATGgtataaacaattctgtaagcattttgcttgtcgaggtgtagtttggaagccatcataaccatgcatttttctt
tctcttctacagGTTAGTTACTGTGAAAATGATGTGGATTGCAGACGTTTACTTCAACTGATACACTTCGG
AGAGACTTTTGATCCATCACATTGTGCAAAAACATGTGATAATTGCAAAAAAGGATTGAGATGGA
TTGAAAAAGATGTGACCAATATTGCCAAGCAATTGgtaggtgcctaagtagatatctgtgccctgtctcctgaacacatgcacacc
tctctgtgaacttacatcatcaccttgttgcttttttagGTCGAGCTGGTCTTGGCGACAGGGCAGCCATGTTCAAGTTCTCA
TATTCTTGAAGTTTACAGGGGTTCCTTGAACCAAAATgtatgcatggtgaaattaaacactcggaattgttgttctgaagttcatta
attctgttacttgctaccagtgtaaacttactttctgcactctattgtgcatcttggcttcttgctggggaaatcatggtgaaactacattcttgccctagattgtaaatcttgt
caacactgcatttagGTCAAGAAGAACCGTCATGATATGTTGCCTCTTCATGGAGCTGGAAAGCATCTAGCT
AAAGGTGAGGCAGCAAGAGTACTACGGAATCTAGTTACTGAGGGAATACTTGCTGAGGATGTCAA
AAAGAGCGATACATATGGATCAGTATCATCTGTTTTAAAGgtagccgaatgaatacttgatccctggttggtttattactttattt
tgagacaattttttggcagccttcttgtaactgccttggtctcatacatgtgtttttgttgagcagGTGAATCAGGTTAAAGTTGGTGGTCTTC
GCTCTGGCAATCAGATCGTCCTTAAgtttgttttctgccttacatgttataaaatttattggatgttctgcatttgcctcttctcttcaagtttaatcga
catatcttgattttattgagaaatatttccttttctgtgtgaccatactattatttttcatgagtttgcctaaaatctttctagggttcatcattaactaagaacgtctttaagtgat
ggttgcacttctaaattggtttgcacaatatgatgttaccgacaatctgatcttggtttgtagaactgaatatatatataatgttaaatgtcattatgatgatttttttggcagtgt
aagttttttgcacaatgtaatgttatcgacaatctgatcttgatttgtagaaccgaatacgtaatgtcaaatgtcattatcgattgagactttagttgaatgcacttcaaattg
ggaagttgagctttaattaaattaggatatcacatactctactagagtactttctttaaatttaaatttcatctaatactccaaacatcttgcttataatgctctgtatgttattttg
cagGTTTCCCACTCCTGACAAGGCCCCTAAGATGGGGAAACTTGACGAATCATCAATCTCACAAGTC
AATAAGCCTGTTCAACGGCAGAGTGAAATGGATGAGgtagttggtcattagtaaatcctcttgtgtatttattttggttcgttctaaag
gctattctcaattgcagAATTTTTCATCGTTGCTCTATGAAACTTTAAGAATCCTTAGGTCTCAGATAGCAGAG
GCTGCAGGATGTGGTGTACACCACATATTTAAgtaagttccttgtcatacctccttgtcactcactgacttctgacgtggtgaattattca
tgttcagTAATGAAACACTGAAGGAAATTAGCACTCGGGTACCAAGGACAAAAGAAGAACTTTTGGAG
ATAAACGGCATCGGCAAgtaatgactctaaaaacaatatgttcagtgcacattgtaaggttgacaatgcagggttgagaactaggacaagaaattta
cttactgttttacataccttctttcagGGTGAAGCTCAACAAGTATGGAGATCGCGTACTTGCAACCTTAGAGGAGTT
TCTCAACCAATTTTCAAGCGGAAGCAAGAGAAGCAGCAGCAGCGGTGGCAGCAACGAGCAAAAC
GAGGCCGTGAAGAAGCGAAGGGGCTTCACCGCCATCGACACCTCTGGGAACGGCAATGACTTTGA
AGAACGCACAGTTCAGTCCAAGAAACACGCAGGAAAGACACAAAACAGCAAGCAGGGAATAGCC
GATGCTGCAAGCGTGATCCAAGACATCCGCTATATAGATGTTGACTTGGACGGATGCGAAGATGT
GGATGAAGAGCTGTGCAGCAGTGTCCAACAGCCCGTGGCCTCTGGTAGGGTTTTGCCCAAGTGGTC
AGCCGGCGGTAATGCCCCCCCACCTAATATATTTGAAGAATTTAAATACACCAACTAG
GCTTGTAGGCATGTAAGCCGTCATATATATAGGTCTCGTGTTCCGGGGTAGTAGTTTTTGTAGGCT
GTAGCGCATGTAACATGGTATTCTTCTGTGGGGAAATCCGGATTCGGCGGGCATGGAAATGTATGT
ACCACATTTGCAGTAAGAGACAAGTGTGTAATTGGAAGTGCC

>RecQl4
MQGSNKLNAGSNCNDKLPKVNWPHHANAIQSSLSKGDFLSSSFLFSLPTQRANPEANCNTMHSLRSAA
CKIQGPERLQVPWIEKAWRSVCNTQVTCKNYLRPGLSAKVKDCGRDFALTYAADSSYNAKKLDNVPK
STILSEGSLHQRTESGILEQNSSHRTCTMAYQSNHVVGTTYQSSFARTDAMSCQTVPVADNMCADDKL
DAMDDDEILASIDVDRIVMEHYEATNTPRGLASRQMSTPSGNKFNFTGLDENSLPQELSEICSHGYKLA
FCPEANYHLQGMKDQLISVSNKLLDGSGELSPQHSEELRQQRAHLNKQIQILGDYMARPTQDDERKRS
HSMASTTAAEGHRPPMTPSTFVDNNRSQSQFYDTNGPWDGGSCYTPAPCPYMDSNIPLTSVQRDYTRR
NIDISYTDGSGDKKWSSTDFPWTKELEVHNKRVFGNRSFRPNQREIINATMNGSDVFVLMPTGGGKSL
TYQLPALIEEGITLVVCPLVSLIQDQIMHLSQANIPATYLSANLEWTEQQRILRDLMSPTSTCNYKLLYV
TPEKIAKSDVLLRQLENLYSRGYLSRIVIDEAHCVSQWGHDFRPDYQHLGVLKQKFPETPVLALTATAT
ASVKEDVVQALGLANCVVFKQSFNRPNLRYFVMPKTKKCLEDIDRFIRENHHKECGIIYCLSRMDCEK
VAEKLREYGHQASHYH[G]NMEPFDRTQVQRLWSMDKINIICATVAFGMGINKPDVRFVIHHSLPKSIE
GYHQECGRAGRDGQRSSCVLYYNYSDYIRVKHMITQGSAEQVRSSSSSSHGQALATHKENLLCMVSY
CENDVDCRRLLQLIHFGETFDPSHCAKTCDNCKKGLRWIEKDVTNIAKQLVELVLATGQPCSSSHILEV
YRGSLNQNVKKNRHDMLPLHGAGKHLAKGEAARVLRNLVTEGILAEDVKKSDTYGSVSSVLKVNQV
KVGGLRSGNQIVLKFPTPDKAPKMGKLDESSISQVNKPVQRQSEMDENFSSLLYETLRILRSQIAEAAG
CGVHHIFNNETLKEISTRVPRTKEELLEINGIGKVKLNKYGDRVLATLEEFLNQFSSGSKRSSSSGGSNE
QNEAVKKRRGFTAIDTSGNGNDFEERTVQSKKHAGKTQNSKQGIADAASVIQDIRYIDVDLDGCEDVD
EELCSSVQQPVASGRVLPKWSAGGNAPPPNIFEEFKYTN*
