# Supplementary figures and images for: An Induced Mutation in HvRECQL4 Increases the Overall Recombination and Restores Fertility in a Barley HvMLH3 Mutant Background
Source: Front Plant Sci. 2021 Nov 12;12:706560. doi: 10.3389/fpls.2021.706560 (PMC8633572; doi:10.3389/fpls.2021.706560)

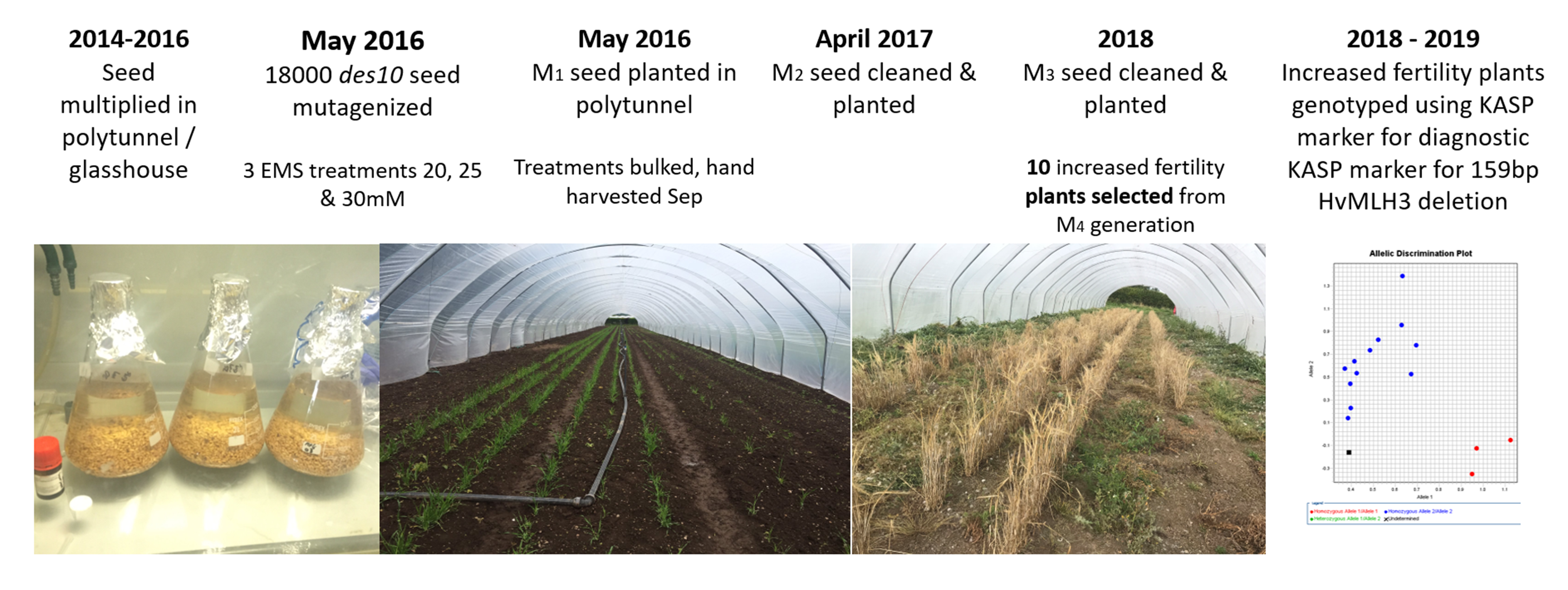

Supplement: Supplementary Figure 1 — Suppressor screening population development and process summary. [file Image_1.tif]

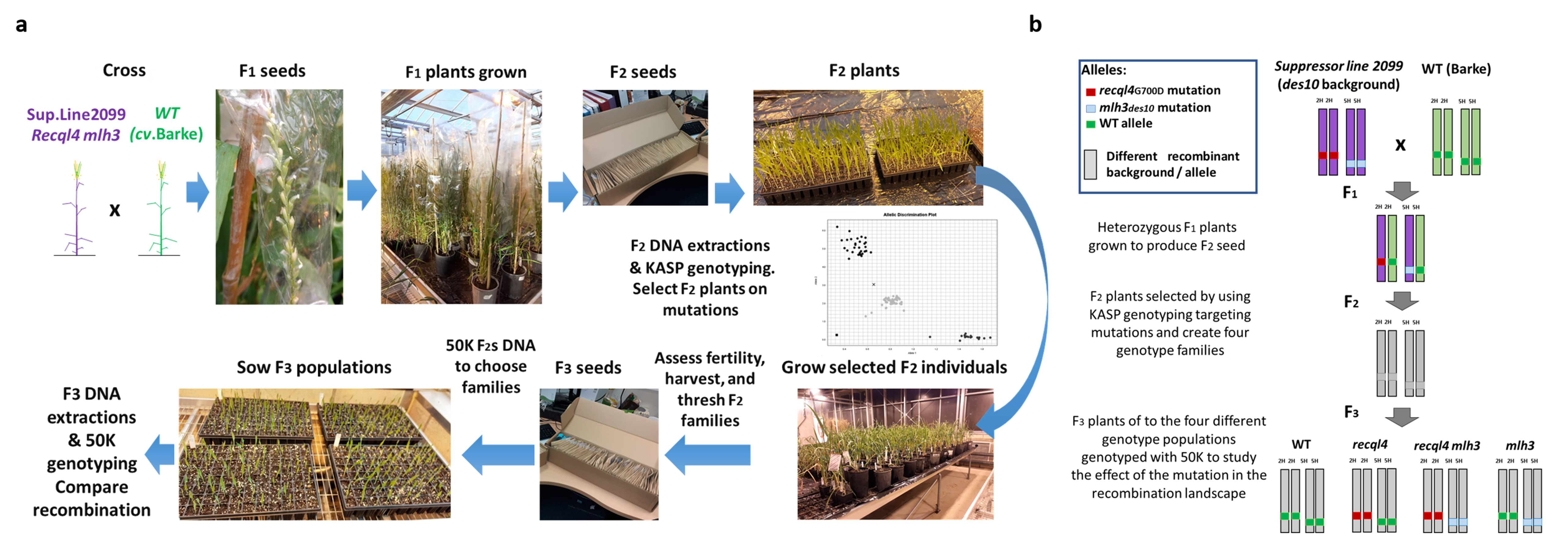

Supplement: Supplementary Figure 2 — (A) Outline of the development of F3 populations to be genotyped. (B) Selection of the genotypes across the generations to create F3 populations. SuppLine2099 was crossed with cv. Barke background to generate heterozygous F1s, which were grown to produce F2 seed. F2 plants were grown and selected by using Kompetitive allele-specific PCR (KASP) genotyping for targeting the segregating mutations. Only the four homozygous combinations were selected to be grown as F2 families and produce F3 seeds. These were later grown and genotyped with the 50K assay to study the effect of the mutation in F2 recombination. The alleles in the mutated genes are indicated by the square box color, being green for the wild type (WT)-like allele, dark red for the recql4G700Dmutation, and light blue for the mlh3des10 mutation. The gray area in the chromosomes indicates different recombinant backgrounds among the populations. [file Image_2.tif]

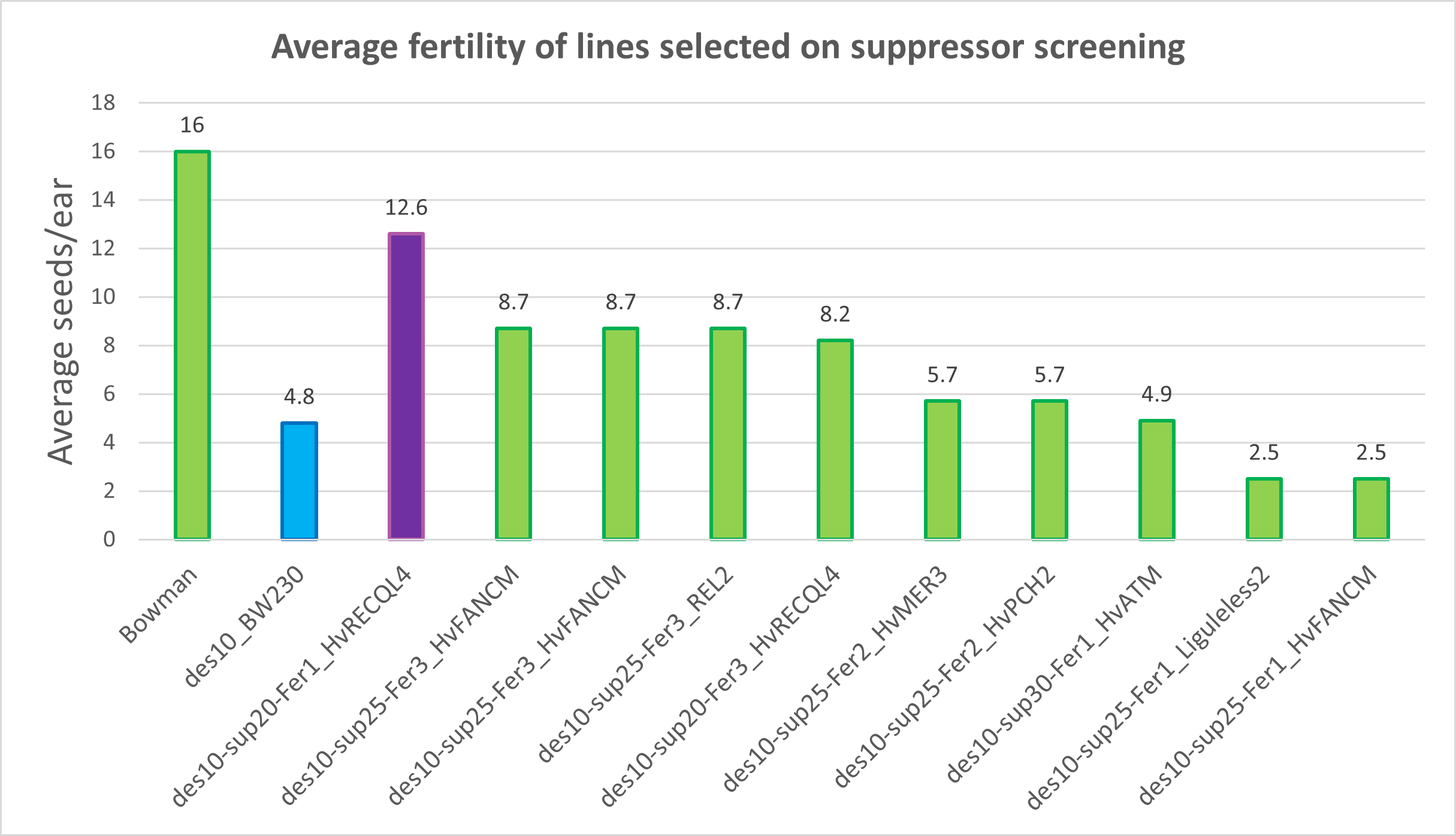

Supplement: Supplementary Figure 3 — Barchart showing the average fertility of the three plants per selected line in the suppressor screening. SuppLine2099 was chosen for the present study It is marked in purple and desynaptic10 (des10) in blue. [file Image_3.tif]

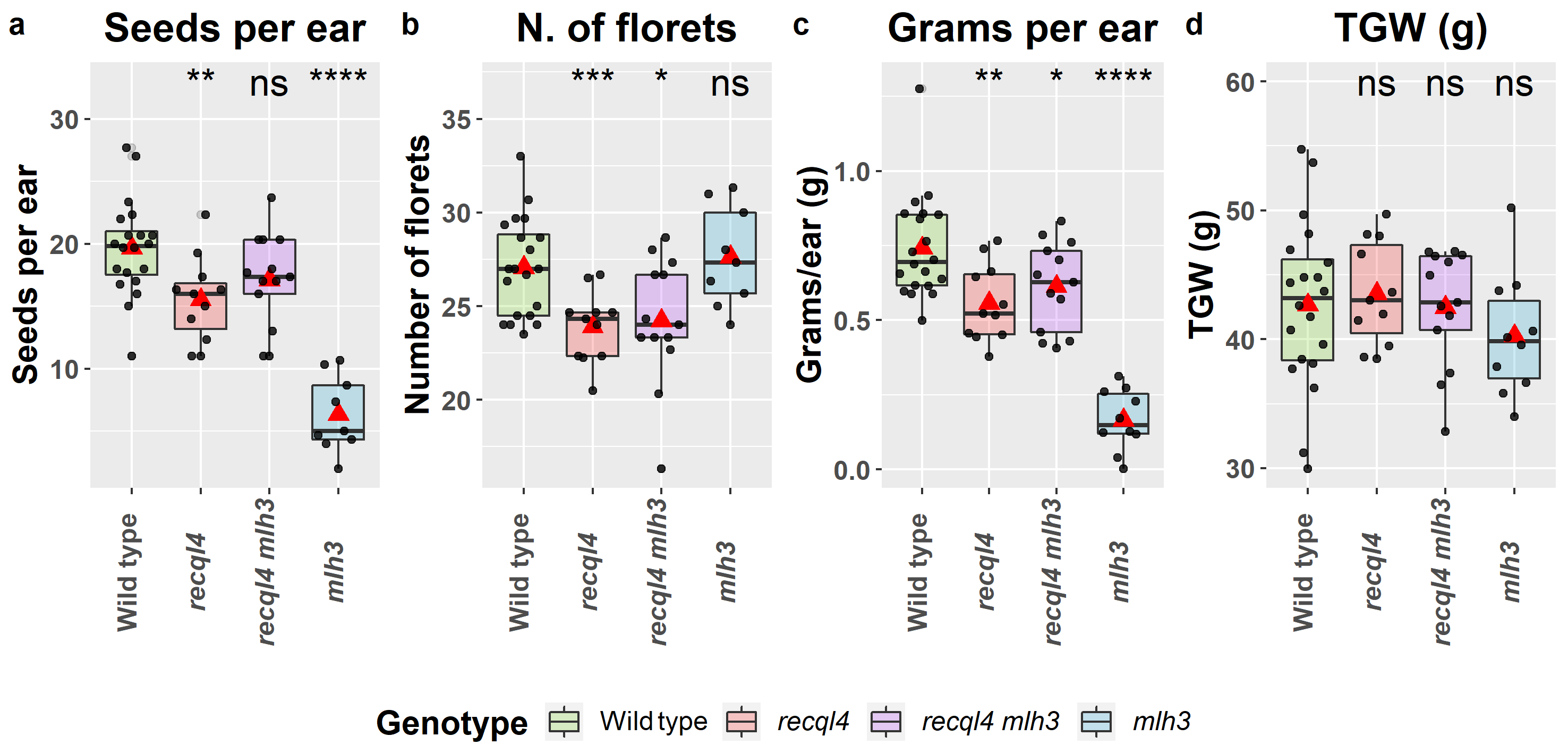

Supplement: Supplementary Figure 4 — Different fertility phenotypes of the selected F2 plants. (A) Total seeds per ear, (B) the Total number of florets per ear, (C) Total grams per ear, (D) Thousand grain weight (TGW). The statistical comparisons were done with the t-test where “ns” is not significant, *p < 0.05, **p < 0.01, ***P < 0.001, ****p < 0.0001. All the comparisons are done by using the WT as a reference. The red triangle shows the mean per genotype. Each dot in the graph represents the average of 3 years of one plant, except for grams per year and TGW, where all the ears of the plants were used. [file Image_4.tif]

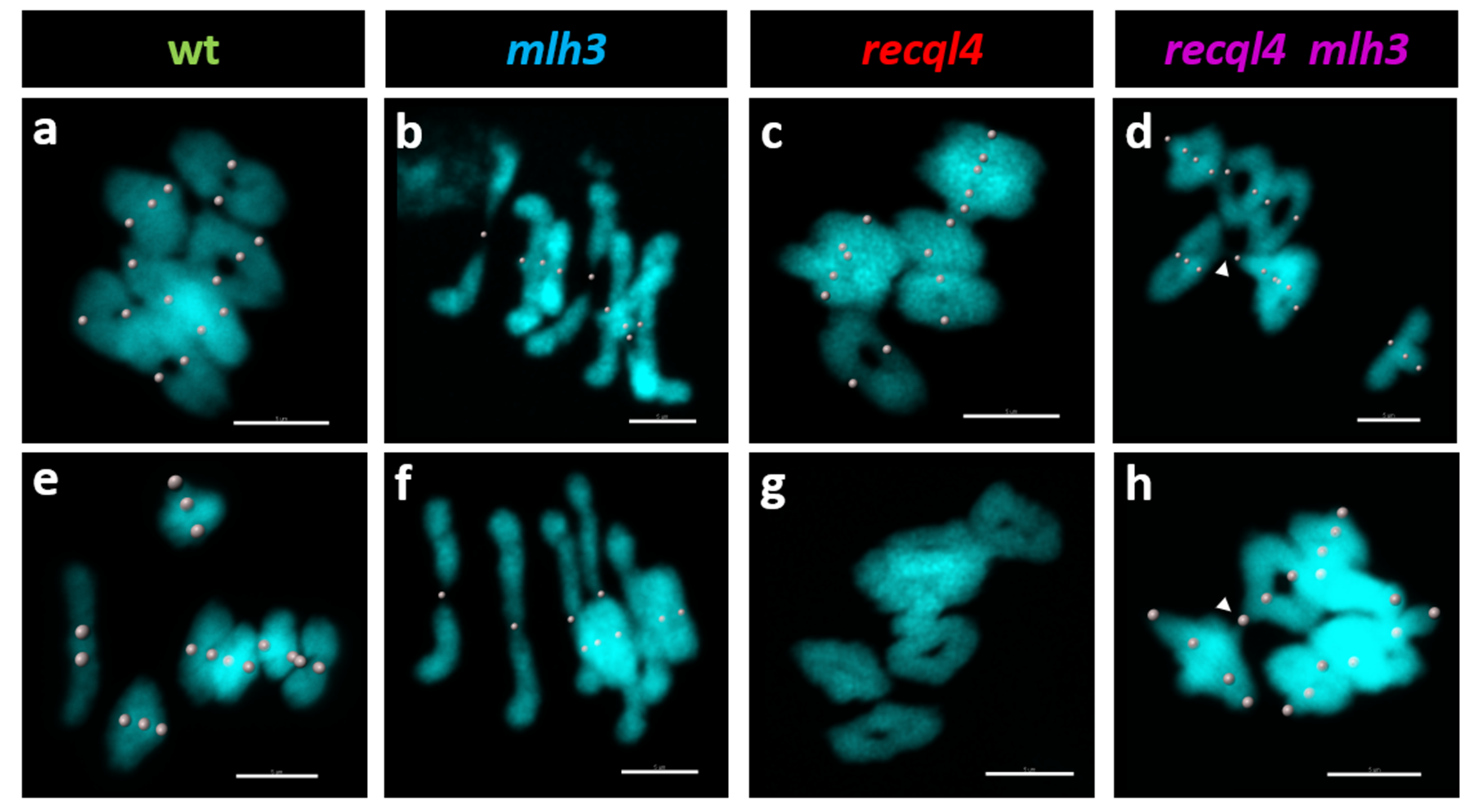

Supplement: Supplementary Figure 5 — Cytology of male meiosis on the F4 plants of different populations: (A,E) WT, (B,F) mlh3, (C,G) recql4, and (D,H) recql4mlh3. Chiasmata counts are pointed with the gray dots (when discernible) and the chromosome bridges marked with white arrows. Bar size: 5 μm. [file Image_5.tif]

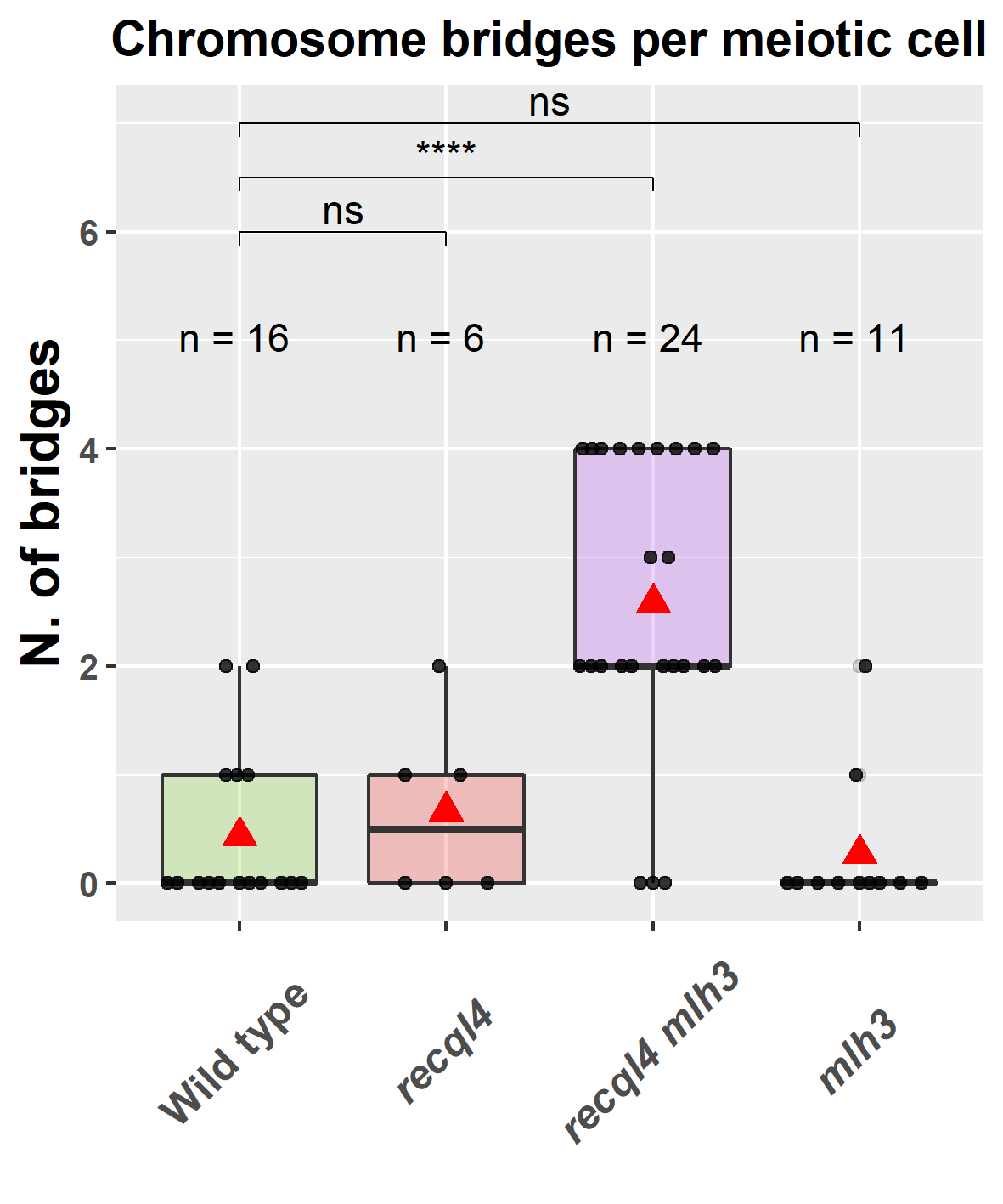

Supplement: Supplementary Figure 6 — Quantitative scoring of the chromosome bridges per meiotic cell. The statistical comparisons are done with the t-test where “ns” is not significant, ****p < 0.0001. All the comparisons are done by using the WT as a reference. [file Image_6.tif]

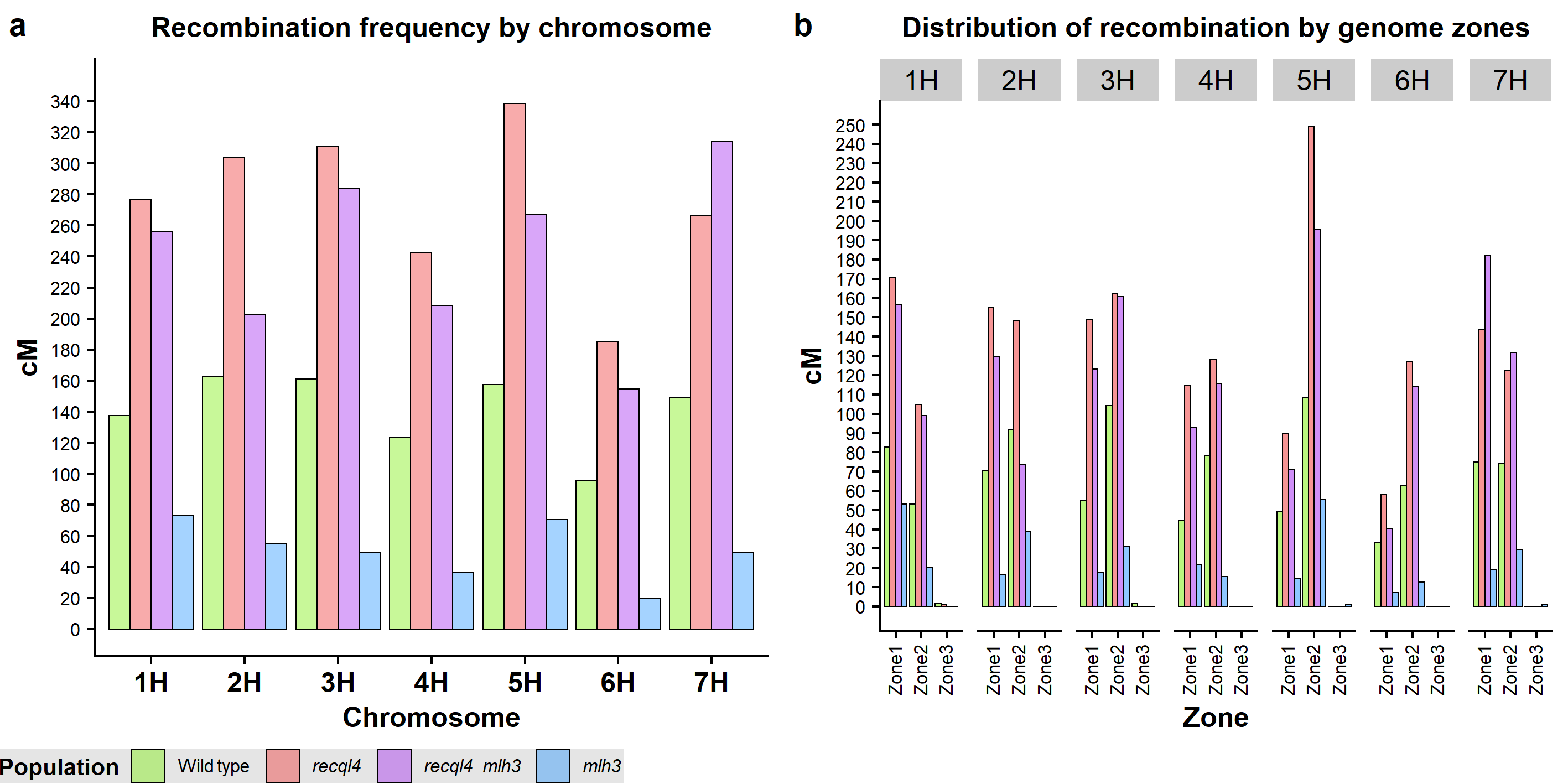

Supplement: Supplementary Figure 7 — (A) Genetic chromosome length (cM) of every chromosome for each population. (B) Distribution of recombination by the genomic zones for each population chromosome-wise. [file Image_7.tif]
